# Glucose transporters and sodium glucose co-transporters cooperatively import glucose into energy-demanding organs in carcinogenic liver fluke *Clonorchis sinensis*

**DOI:** 10.1371/journal.pntd.0012315

**Published:** 2024-07-05

**Authors:** Fuhong Dai, Soon-Ok Lee, Jin-Ho Song, Won-Gi Yoo, Eun-Hee Shin, Xuelian Bai, Sung-Jong Hong

**Affiliations:** 1 Department of Parasitology, School of Biology and Basic Medical Sciences, MOE Key Laboratory of Geriatric Diseases and Immunology, Suzhou Key Laboratory of Pathogen Bioscience and Anti-infective Medicine, Suzhou Medical College, Soochow University, Suzhou, China; 2 Department of Medical Sciences, Chung-Ang University College of Medicine, Seoul, Republic of Korea; 3 Department of Medical Zoology and Medical Research Center for Bioreaction to Reactive Oxygen Species and Biomedical Science Institute, School of Medicine, Graduate School, Kyung Hee University, Seoul, Republic of Korea; 4 Department of Pharmacology, Chung-Ang University College of Medicine, Seoul, Republic of Korea; 5 Laboratory of Veterinary Parasitology, College of Veterinary Medicine and Research Institute for Veterinary Science, Seoul National University, Seoul, Republic of Korea; 6 Department of Tropical Medicine and Parasitology, Seoul National University College of Medicine, and Institute of Endemic Diseases, Seoul, Republic of Korea; 7 Seoul National University Bundang Hospital, Seongnam, Republic of Korea; 8 Medical Research Center, Binzhou Medical University Hospital, Binzhou, China; 9 Center for Infectious Vectors and Diseases, Incheon National University, Incheon, Republic of Korea; Aberystwyth University - Penglais Campus: Aberystwyth University, UNITED KINGDOM OF GREAT BRITAIN AND NORTHERN IRELAND

## Abstract

**Background:**

The liver fluke *Clonorchis sinensis* imports large amounts of glucose to generate energy and metabolic intermediates through glycolysis. We hypothesized that *C*. *sinensis* absorbs glucose through glucose transporters and identified four subtypes of glucose transporter (CsGTP) and one sodium glucose co-transporter (CsSGLT) in *C*. *sinensis*.

**Methodology/Principal findings:**

Expressed sequence tags encoding CsGTPs were retrieved from the *C*. *sinensis* transcriptome database, and their full-length cDNA sequences were obtained by rapid amplification of cDNA ends (RACE). The tissue distribution of glucose transporters in *C*. *sinensis* adults was determined using immunohistochemical staining. Developmental expression was measured using RT-qPCR. The transport and distribution of glucose into living *C*. *sinensis* were monitored using confocal microscopy. Membrane topology and key functional residues of CsGTPs were homologous to their counterparts in animals and humans. *CsGTP1*, *2*, and *4* were transcribed 2.4–5.5 times higher in the adults than metacercariae, while *CsGTP3* was transcribed 2.1 times higher in the metacercariae than adults. *CsSGLT* transcription was 163.6 times higher in adults than in metacercariae. In adults, CsSGLT was most abundant in the tegument; CsGTP3 and CsSGLT were localized in the vitelline gland, uterine wall, eggs, mesenchymal tissue, and testes; CsGTP4 was found in sperm and mesenchymal tissue; and CsGTP1 was mainly in the sperm and testes. In *C*. *sinensis* adults, exogenous glucose is imported in a short time and is present mainly in the middle and posterior body, in which the somatic and reproductive organs are located. Of the exogenous glucose, 53.6% was imported through CsSGLT and 46.4% through CsGTPs. Exogenous glucose import was effectively inhibited by cytochalasin B and phlorizin.

**Conclusions/Significance:**

We propose that CsSGLT cooperates with CsGTPs to import exogenous glucose from the environmental bile, transport glucose across mesenchymal tissue cells, and finally supply energy-demanding organs in *C*. *sinensis* adults. Studies on glucose transporters may pave the way for the development of new anthelmintic drugs.

## Author summary

The Chinese liver fluke *C*. *sinensis* is a fish-borne trematode that causes serious biliary diseases and complications, such as cholangiocarcinoma. Living in an anaerobic environment, *C*. *sinensis* adults consume large amounts of exogenous glucose and produce energy and intermediate products via glycolytic pathways. We identified four glucose transporters (CsGTP1–4) and one sodium glucose co-transporter (CsSGLT) in *C*. *sinensis* and their glucose transport mechanism was investigated. This is the first study to report SGLT in a helminthic parasite, particularly trematodes. Elucidating the structure and function of CsGTPs, CsSGLT, and their inhibitors may lead to strategies to exhaust glucose and ultimately starve *C*. *sinensis*. This study provides fundamental information on the glucose transporters of helminths and may shed light on directions for studying energy metabolism in other mammalian-infecting trematodes.

## Introduction

*Clonorchis sinensis* metacercariae excyst in the duodenum, migrate by bile chemotaxis, and inhabit the bile ducts of mammalian hosts [1–3]. Adult flukes produce excretory and secretory products that damage the biliary epithelium and cause bile duct fibrosis. Common symptoms of human infections include fatigue, obstructive jaundice, abdominal pain, and indigestion. Chronic clonorchiasis can cause complications including cholecystitis, cholelithiasis, biliary cirrhosis, pyogenic cholangitis, and cholangiocarcinoma [4].

Glucose is an essential nutrient and energy source for living organisms, and parasites are no exception. Parasitic flukes require large amounts of energy for their growth, movement, and reproduction. *C*. *sinensis* inhabits an anaerobic environment and generates energy via glycolysis. An earlier study traced ^14^C-glucose metabolism in *C*. *sinensis* using glycolytic enzymes, phosphorylated intermediates of glycolysis, and labeled glucose end products [5]. *Fasciola hepatica* adults also utilize glucose as an energy source via glycolysis [6]. *Schistosoma mansoni* adults consume glucose equivalent to their dry body weight every five hours [7]. Thus, for trematodes to survive in anaerobic environments, they must be continuously supplied with large quantities of glucose, their main energy source. Trematodes absorb glucose from the environment via specialized transporters. Common mechanisms of sugar transport in eukaryotes include facilitated transport and active transport, often coupled with ion movement using ATP [8].

Glucose transporters (GTP) have been characterized in parasites. Two facilitative hexose transporters have been discovered in *Trypanosoma brucei*: THT1 and THT2, which are differentially promoted in the bloodstream and procyclic forms of the parasite, respectively [8–10]. *Trypanosoma cruzi* and *T*. *vivax* possess the facilitative hexose transporters TcrHT1 and TvHT1, respectively [10]. A facilitative hexose transporter (PfHT) is essential for the survival of the erythrocytic stages of *Plasmodium falciparum* [11]. In the tapeworm *Taenia solium* within the phylum Platyhelminthes, two facilitative glucose transporters, TGTP1 and TGTP2, have been identified [12]. Four facilitative glucose transporters (SGTP1–4) have been reported in *S*. *mansoni*. Among these, SGTP1, 3, and 4 were successfully cloned; however, SGTP2 was identified as a pseudogene because of an incomplete open reading frame (ORF) blocked by a stop codon at the N-terminus. Glucose transport by SGTP is inhibited by the GTP inhibitor cytochalasin B [13]. SGTP4 and SGTP1 are respectively localized to the apical surface and the basal membrane of the tegument [14,15]. Silencing of *SGTP1* and *SGTP4* impairs glucose uptake in blood flukes and increases mortality [16].

Two types of glucose transporters are coupled with ion transport: the glucose/Na^+^ co-transporter (SGLT) and the glucose/H^+^ co-transporter. In the mammalian intestine, two membrane proteins, SGLT and the Na^+^/K^+^-ATPase, function together to transport glucose along an energy-dependent Na^+^ gradient. Glucose/H^+^ transporters are primarily found in prokaryotes, particularly *Escherichia coli*. H^+^-ATPase or the respiratory chain, contributes to the generation of a proton gradient [8]. These glucose transporters allow sugar absorption, fulfilling the basic physiological needs of individual organisms [17].

Based on this information, we hypothesized that *C*. *sinensis* absorbs large amounts of glucose through glucose transporters as an energy source for glycolysis. In the present study, we report the presence of four GTPs and one SGLT in *C*. *sinensis* and their molecular and functional characteristics.

## Methods

### Ethics statement

Rabbits (New Zealand white, female, 2.3 kg; Koatech, Seoul, Korea) and mice (BALB/c, female, 7 weeks old; Orient Bio Inc., Gyeonggi-do, Korea) were handled in an accredited Chung-Ang University animal facility (Korea FDA-accredited unit; unit number 36), in accordance with the Association for Assessment and Accreditation of Laboratory Animal Care International Animal Care policy. All animal experiments were approved by the Institutional Review Board of the Chung-Ang University Animal Facility (approval number CAU-2011-0052 and CAU-2011-0013).

### cDNA clones of CsGTPs and CsSGLT

Clusters/expressed sequence tags (ESTs) annotated as “glucose transporter” were searched and retrieved from the *C*. *sinensis* transcriptome database in the Korea Disease Control and Prevention Agency [18]. We found seven clusters and one EST encoding glucose transporters, and one cluster encoding a sodium glucose co-transporter (S1 Table). All ESTs in the nine clusters were multiple-aligned according to GTP subtypes, and a master EST clone containing the 5′-end closest to the first methionine codon was selected from each of the five subtype GTPs: CSA24824, CSE02055, CSA17430, CSA07662, and CSA17668 (S1 and S2 Figs). Glycerol stocks of these EST clones were retrieved from the *C*. *sinensis* EST library bank of the Korea Disease Control and Prevention Agency. A *C*. *sinensis* cDNA library was constructed using pBK-CMV. Selected EST clones were cultured in Luria–Bertani medium containing kanamycin (30 μg/mL). The plasmid DNA containing EST cDNA was extracted using a QIAprep Spin Miniprep Kit (QIAGEN, Hilden, Germany). ESTs were sequenced to obtain the 3′-end of each cDNA. The EST cDNA sequences were subjected to BLAST analysis on the NCBI NLM website (http://blast.ncbi.nlm.nih.gov/) revealing their homology to the GTP gene family.

Genomic DNA, hypothetical cDNA, and deduced polypeptide sequences homologous to partial CsGTP EST sequences were retrieved from the NCBI database. When compared, the master ESTs were found to be missing certain segments in the 5′-region. To obtain the 5′-end of the CsGTP cDNA, 5′-Rapid Amplification of cDNA Ends (RACE) was performed using the total cDNA of adult *C*. *sinensis*. Forward primers were designed to target the cDNA stretches containing the first methionine of each hypothetical CsGTP. Amplicons were direct-sequenced using their respective 5′-RACE primers. Once linked to the secured 5′-sequence, CsGTP extended the cDNA stretch, encoding the entire polypeptide (S1–S3 Figs) [19]. The genomic organization, exons, and introns of CsGTPs were analyzed to determine the cis- and/or trans-splicing of RNAs, as described previously [19].

### cDNA sequence and protein structure

Multiple alignments of CsGTPs with glucose transporter proteins from different species in GenBank (http://www.ncbi.nlm.nih.gov) were created using the CLUSTAL X program (EBI, Cambridge, UK). The GTP phylogenic tree of *C*. *sinensis* and other species was drawn using MEGA v5.0 (http://www.megasoftware.net/).

Secondary structures and transmembrane domains (TM) were predicted using TMpred (http://www.ch.embnet.org/software/TMPRED_form.html) and verified using TMHMM v2.0 [20]. The transmembrane helix number, location, loop length, adjacent motif structures, and surrounding sequence characteristics were determined using SWISS-PROT (http://web.expasy.org/docs/swiss-prot_guideline.html).

### Subcloning the antigenic regions of CsGTPs and CsSGLT into bacterial expression vector

B-cell epitopes were predicted using the BepiPred linear epitope prediction program (http://tools.immuneepitope.org/tools/bcell/iedb_input) to produce the antigenic chimeric proteins of CsGTP1, 3, 4, and CsSGLT. The hydrophilic regions of CsGTPs and CsSGLT were predicted using ProtScale (http://web.expasy.org/cgi-bin/protscale/protscale.pl). Two overlapping regions with high epitope probability and hydrophilicity were selected for the chimeric fusion protein (S4 Fig) [21]. Two pairs of PCR primers were designed for both ends of the selected region, and endonuclease restriction sites *Bam*HI and *Hind*III were added to both ends. The corresponding cDNA was amplified using PCR with the Ex Taq DNA polymerase (TaKaRa Bio, Shiga, Japan). The thermal cycle was run with denaturing at 94°C for 30 s, annealing at 58°C for 30 s, and extension at 72°C for 1 min. PCR amplicons were purified using a QIAquick PCR Purification Kit (QIAGEN).

The PCR-amplified cDNA fragments were subcloned into the pCR2.1-TOPO vector (Invitrogen, Carlsbad, CA, USA). Insert-positive colonies were then confirmed by colony-PCR using M13 forward (5′-GTAAAACGACGGCCAGT-3′) and reverse primers (5′-GCGGATAACAATTTCACACAGG-3′), and AccuPower RT PreMix (Bioneer, Seoul, Korea).

The recombinant pCR2.1-TOPO plasmid DNA was double digested with two restriction enzymes, and the inserted cDNA was excised. The bacterial expression vector, pET28a, was double digested using *Bam*HI and *Hind*III endonucleases (Roche, Mannheim, Germany). The insert cDNA was ligated into pET28a using T4 DNA ligase (TaKaRa Bio) overnight at 4°C. The ligated plasmid DNA was transformed into *E*. *coli* DH5α competent cells (Invitrogen) via heat shock at 42°C for 30 s, spread onto LB/kanamycin (30 μg/mL) plates, and cultured overnight at 37°C. The positive colonies were then inoculated into liquid LB medium. Plasmid DNA was extracted and sequenced (Macrogen, Seoul, Korea) using T7 promoter primers. The inserted cDNA was confirmed to maintain the correct sequence and encode the target antigenic peptide [19,21]. For the CsSGLT immunogenic chimeric protein, B-cell epitopes were selected and subcloned into the expression plasmid pET28a using the same method as described above for CsGTP subcloning.

### Expression and purification of chimeric CsGTP1, 3, 4, and CsSGLT proteins

The recombinant pET28a plasmid DNA was transformed into *E*. *coli* BL21[DE3]pLysS (Novagen, San Diego, CA, USA) via heat shock at 42°C for 30 s. The transformed bacteria were spread onto LB/kanamycin (30 μg/mL) plates and incubated overnight at 37°C. A colony from the transformed plate was inoculated into the LB medium and incubated with shaking at 37°C until the A600 value reached 0.6. Subsequently, isopropyl β-d-1-thiogalactopyranoside (TaKaRa) was added at a final concentration of 1 mM and incubation continued for another 3 h. *E*. *coli* in the culture were harvested by centrifugation at 4,000 rpm for 10 min at 4°C.

For chimeric CsGTP1, 3, and 4 proteins, *E*. *coli* cells were lysed by sonication on ice in lysis buffer (1% sarkosyl, 1× PBS, pH 7.4, 1% Triton X-100) and then centrifuged at 13,000 rpm for 10 min at 4°C. The supernatant was dialyzed in 1× PBS containing 1% SDS, loaded onto an Ni-NTA column (QIAGEN), and washed with 1× PBS containing 20 mM imidazole 3–5 times until A280 < 0.01 to wash off nonspecific bindings. The chimeric CsGTP1, 3, and 4 proteins were eluted six times with 1× PBS containing 200 mM imidazole (S5 Fig).

The chimeric CsSGLT protein was produced at significant levels in *E*. *coli*. *E*. *coli* cells were lysed by sonicating for 1 min on ice in buffer B (8 M urea, 0.1 M sodium phosphate, 0.01 M Tris-HCl, pH 8.0) containing 10 mM imidazole, then loaded onto an Ni-NTA column, and washed with buffer C (8 M urea, 0.1 M sodium phosphate, 0.01 M Tris-HCl, pH 6.3) 3–5 times until A280 < 0.01. The chimeric CsSGLT was eluted with buffer C containing 150, 200, or 250 mM imidazole. The collected protein eluates were dialyzed against urea-gradient (8, 4, 2, and 1 M) PBS, and finally against 1× PBS containing 1× Complete Mini (Roche) (S5 Fig).

### Production of mouse immune sera

The chimeric antigen proteins were separated on 12% gradient gels (Gradient PAGE Analysis Kit; ELPIS-BIOTECH, Seoul, Korea). After staining with Coomassie Blue (Sigma-Aldrich, St. Louis, MO, USA), the band of interest was excised and equilibrated with sterile PBS three times for 30 min each. Gel slices were homogenized using a tissue grinder with a minimal volume of PBS. The CsGTP or CsSGLT homogenate was mixed with an equal volume of complete Freund’s adjuvant (Sigma-Aldrich) and injected into the abdominal cavity of a BALB/c mouse. Two weeks later, the homogenate was mixed with an incomplete Freund’s adjuvant (Sigma-Aldrich) and injected into the opposite side of the abdominal cavity. After another two weeks, 50 μL of the eluted chimeric protein was injected into the tail vein of the mouse. Three days after the final booster, one or two drops of blood were collected from the mouse tail, and antigen-specific antibodies were confirmed using western-blotting. When the production of a specific antibody was verified, the mouse was euthanized and the serum was saved (S5 Fig).

### Preparation of the *C*. *sinensis* metacercariae and adults

*Pseudorasbora parva*, a second intermediate fish host of *C*. *sinensis*, was ground and digested in artificial gastric juice [40 g pepsin 1:10,000 (MP Biochemicals Co., Solon, OH, USA) and 40 mL of concentrated HCl in 5 L of water] at 37°C for 2 h [22]. Rough matter was removed from the digested contents by filtration through a 212-μm mesh sieve. *C*. *sinensis* metacercariae were collected using sieves of 106 and 53 μm diameter meshes and washed thoroughly several times with 0.85% saline solution. Metacercariae were collected from sediments under a dissecting microscope and stored at 4°C until further use.

Rabbits (New Zealand White) were infected with 300 metacercariae and reinfected with the same number of metacercariae one week later. Several months later, the rabbits were euthanized, and *C*. *sinensis* adult flukes were recovered from the bile duct. Infected livers from other rabbits were resected and fixed with 10% neutral formalin.

Adult flukes were thoroughly homogenized using a Tissue-Tearor (BioSpec Products Inc., Bartlesville, OK, USA) on ice in two volumes of PBS (pH 7.4) containing 1% Triton X-100 and 1× Complete Mini. The homogenate was kept at 4°C overnight, centrifuged at 20,000 × *g* for 60 min at 4°C. The supernatant was stored as a soluble extract. The pellet was resuspended in an initial volume of lysis buffer (50 mM Tris-HCl, pH 8.0, 150 mM NaCl, 0.02% sodium azide, 1% NP-40, and 1 mM PMSF) and placed on ice for 30 min. After centrifugation as above, the supernatant was collected as the insoluble extract.

### Immunoblotting

The chimeric CsGTPs, CsSGLT, and a soluble extract/antigen of *C*. *sinensis* adult flukes were separated by 12% SDS-PAGE and transferred onto a nitrocellulose membrane (Amersham Hybond ECL, GE Healthcare Bio-Sciences, Uppsala, Sweden). Target proteins on nitrocellulose membranes were detected using mouse immune sera at a 1:200 dilution. The secondary antibody, goat anti-mouse IgG AP-conjugated (Sigma-Aldrich) was used at 1:5,000 dilution. Color was developed using a BCIP/NBT substrate (Sigma-Aldrich).

### Immunohistochemical staining

*C*. *sinensis* adult flukes in rabbit livers were fixed in 10% neutral formalin and processed into paraffin blocks. The paraffin-embedded sectioned flukes were deparaffinized, rehydrated, and incubated in mouse immune serum at a 1:200 dilution for 2 h at room temperature (22–24°C). The ribbons were incubated with a polymer-horseradish peroxidase-labeled anti-mouse IgG system at 1:1,000 dilution (Dako Cytomation EnVision, Agilent, Santa Clara, CA, USA). Color was developed using a 3-amino-9-ethylcarbazole substrate, and counterstained with hematoxylin.

### Quantitative analysis on *CsGTP* and *CsSGLT* gene expression

*C*. *sinensis* adults and metacercariae were frozen in liquid nitrogen and pulverized using a mortar and pestle. TRIzol reagent (Invitrogen, Carlsbad, CA, USA) 1 mL was added to 200 μL of *C*. *sinensis* powder and centrifuged at 12,000 rpm for 10 min. One milliliter of chloroform was added to the supernatant, mixed thoroughly, and centrifuged at 15,000 rpm for 15 min. The aqueous phase was then removed and isopropanol was added to precipitate RNA. The total RNA was pelleted by centrifugation and washed with 75% ethanol. The RNA pellet was dissolved in 0.1% DEPC-treated distilled water, and trace DNA was removed using a DNase-free kit (Ambion, Austin, TX, USA). The total RNA quality and concentration were measured spectrophotometrically at OD_260_/OD_280_. To determine the purity, total RNA was run on a 1% formaldehyde-agarose gel and the presence of ribosomal RNA bands confirmed the quality of the RNA. First-strand cDNA was synthesized from 1 μg of total RNA using the Mazinme RT PreMix Kit and oligo(dT) primer (Intron, Sungnam, Korea) at 45°C for 60 min. The synthesized cDNA was stored at −20°C until use.

For PCR amplification, forward and reverse primer sets (Table 1) specific to the target gene transcripts of CsGTPs and CsSGLT were designed using Oligo Primer ver. 6.7 (Molecular Biology Insights, Cascade, WA, USA). The PCR primers were synthesized by Bioneer Co., Ltd. (Daejeon, Korea). For the relative comparison of gene expression between the developmental stages of *C*. *sinensis*, β-actin, calcyphosine, and phosphoglycerate kinase genes were used as reference standards [23].

**Table 1:** Primers for *CsGTP1*, *2*, *3*, *4*, and *CsSGLT* for RT-qPCR.

Gene	Primers	
*CsGTP1*	Forward	5′-TGAGAAAAAAGGGCGACGAAC-3′
	Reverse	5′-CAAGGAAGGGAAGGAAGGAGT-3′
*CsGTP2*	Forward	5′-GCTTTCTTCTGCCTTATCA-3′
	Reverse	5′-AGATAAAAGATTGCCTGACC-3′
*CsGTP3*	Forward	5′-TTTCTGATTGGCGGTCT-3′
	Reverse	5′-AGGACTTCTTAGGCGTTGGAG-3′
*CsGTP4*	Forward	5′-TGGAGGTGGCGAAAAATCAA-3′
	Reverse	5′-AGAAGTGAAGCAGCAAGAAC-3′
*CsSGLT*	Forward	5′-CCTATGGGGCTTTGATGTCT-3′
	Reverse	5′-CGAGTTTCCTTGGCTTGTACC-3′

For quantitative PCR (qPCR), 50 ng each of first-strand cDNA from *C*. *sinensis* adults or metacercariae were mixed with 0.5 μM of each forward and reverse primer and 1× SYBR Green master mixture in a final volume of 10 μL (Roche, Mannheim, Germany). DNA polymerase was activated in the reaction mix at 95°C for 15 min. Target gene transcripts were amplified using a PCR machine (PRISM 7000 PCR system, ABI, Foster, CA, USA) with 40 thermal cycles of 10 s at 95°C and 10 s at 60°C. To check the specificity of the primers, dissociation curves were plotted by running one thermal cycle of 95°C for 10 s, 65°C for 60 s, and 0.1°C/s increment to 95°C. Threshold cycle values (C_T_) of the target and reference genes were obtained using Sequence Detection Software ver. 1.2.3 (ABI). Relative differences of target genes between developmental stages were calculated using the 2^−ΔΔCT^ method [23].

### *In vivo* glucose uptake assay by confocal microscopy

Adult *C*. *sinensis* were starved overnight in a maintenance solution (140 mM NaCl, 5 mM KCl, 2.5 mM CaCl_2_, 1 mM MgSO_4_, 1 mM KH_2_PO_4_, and 10 mM HEPES, pH 7.4). After washing three times with 0.01 M PBS, *C*. *sinensis* adults were incubated in maintenance solution containing 1, 2, and 4 mM 2-[*N*-(7-nitrobenz-2-oxa-1,3-diazol-4-yl)amino]-2-deoxy-d-glucose (2-NBDG), an exogenous fluorescent glucose analog (Invitrogen) for 1.25, 2.5, 5, 10, 20, and 40 min, respectively. The flukes were fixed in 0.5% paraformaldehyde for 1 h and washed three to five times. The treated flukes were then mounted onto a glass slide on a Gel Mount (Biomeda Corp., Foster City, CA, USA) with cover glass and covered with aluminum foil to block light. The prepared specimens were observed under an LSM 510 Meta (Carl Zeiss, Heidelberg, Germany) with an excitation of 488 nm and emission of 505–550 nm for 2-NBDG. Image processing and florescence intensity-related data processing were operated in the Image Processing mode of the LSM 510 software. Each organ or region to be analyzed was randomly selected in this mode, and the florescence intensity of each selected region was automatically converted to mean intensity data.

### Statistical analysis

Data were analyzed using the Student’s *t*-test, and a value of *p* < 0.05 was considered statistically significant. All values represent mean ± standard deviation. Means were calculated from independent triplicate assays.

## Results

### Facilitative glucose transporter family in *C*. *sinensis*

In the *C*. *sinensis* transcriptome database, 66 ESTs belonging to seven contigs (CL272Contig1, CL353Contig1, CL1676Contig1, CL1983Contig1, CL2607Contig1, CL3450Contig1, and CL3618Congtig1) and one singlet (CSA24824) were identified as encoding CsGTPs (S1 Table and S6 Fig). Through multiple alignments, ESTs encoding CsGTPs were again grouped into four subtypes (S1 Fig). The whole span of the master cDNA sequence of each subtype of CsGTP was obtained by 5′- or 3′- RACE and confirmed by PCR-walking on the total cDNA of *C*. *sinensis* adults. Full-length cDNA and deduced polypeptide sequences of CsGTP1, 2, 3, and 4 were obtained. CsGTP1, 2, 3, and 4 encode polypeptides consisting of 526, 501, 506, and 496 amino acids, respectively (S7–S10 Figs). The full-length sequences of CsGTPs were registered in NCBI under accession numbers OR678198–678201.

The four subtypes of CsGTP cDNA sequences experimentally validated in this study were compared to their hypothetical CsGTP cDNA sequences retrieved from GenBank (S3 Fig). The retrieved cDNA sequences were GAA56139 for CsGTP1, GAA51825 for CsGTP2, GAA50925 for CsGTP3, and GAA56140 for CsGTP4. CsGTP1 starts from the second exon of GAA56139, and thereafter the two polypeptide sequences were entirely identical. Thus, the hypothetical cDNA GAA56139 contains an additional exon absent in native CsGTP1 cDNA.

The CsGTP2 polypeptide showed identity in two stretches (aa 1–35 and aa 43–308) of GAA51825. Compared to CsGTP2, the hypothetical GAA51825 lacks an N-terminus and has an extra C-terminal stretch. This finding indicated that GAA51825 is a pseudotranscript rather than a functional mRNA. The homolog GAA50925, which is 2.5 times longer than CsGTP3, had two identical stretches (aa 131–563 and aa 753–831) but also had two long additional stretches at both the N- and C-termini. This finding indicated that GAA50925 is hypothetical and does not exist. CsGTP4 showed 100% sequence homology with the hypothetical clone GAA56140 (S3 Fig).

**Fig 1:**
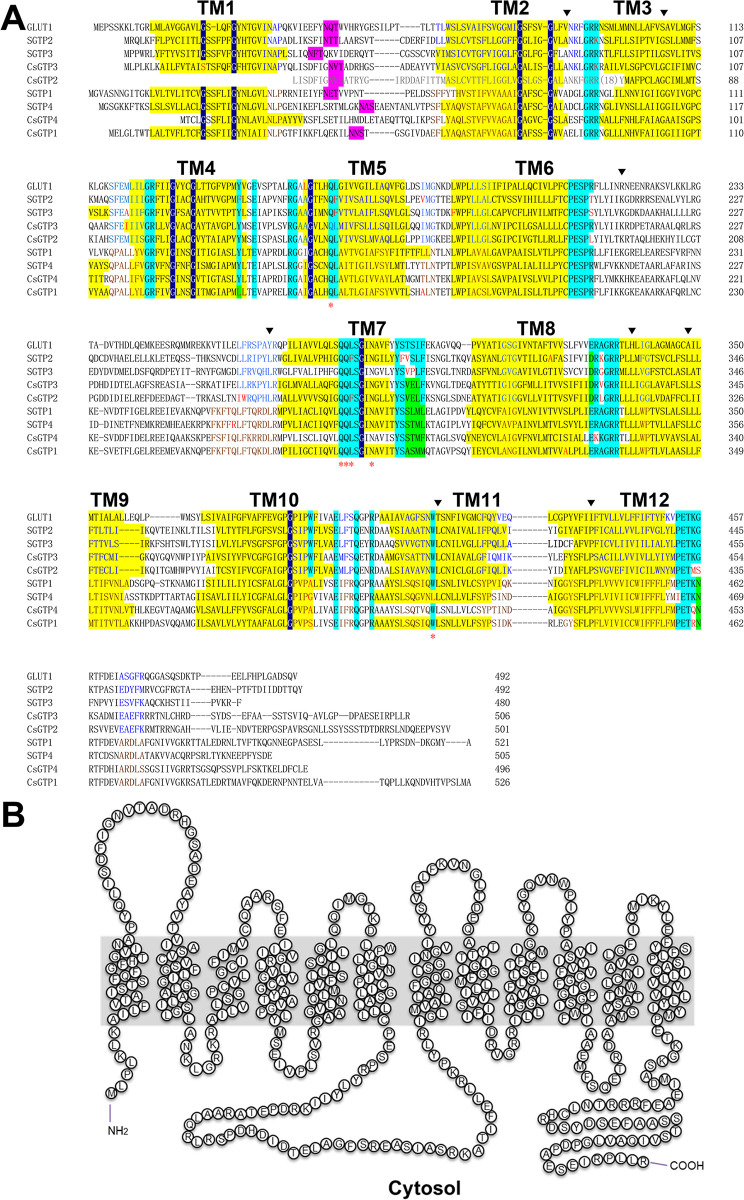
Putative polypeptide sequence and membrane topology of *C*. *sinensis* glucose transporters (CsGTPs). (A) Multiple alignment of CsGTP1, 2, 3, and 4 with *Schistosoma mansoni* GTPs (SGTP1–4) and human glucose transporter 1 (GLUT1). Red asterisk indicates the glucose binding site. Inverted triangles represent amino acids that are conserved in trematodes but differ from human GLUT1. Pink and yellow backgrounds represent the N-glycosylation site and transmembrane domain (TM), respectively. Cyan and black backgrounds represent conserved motifs and glycine residues, respectively. (B) Putative membrane architecture of CsGTP3 showing the 12 TMs predicted using TMpred and TMHMM.

**Fig 2:**
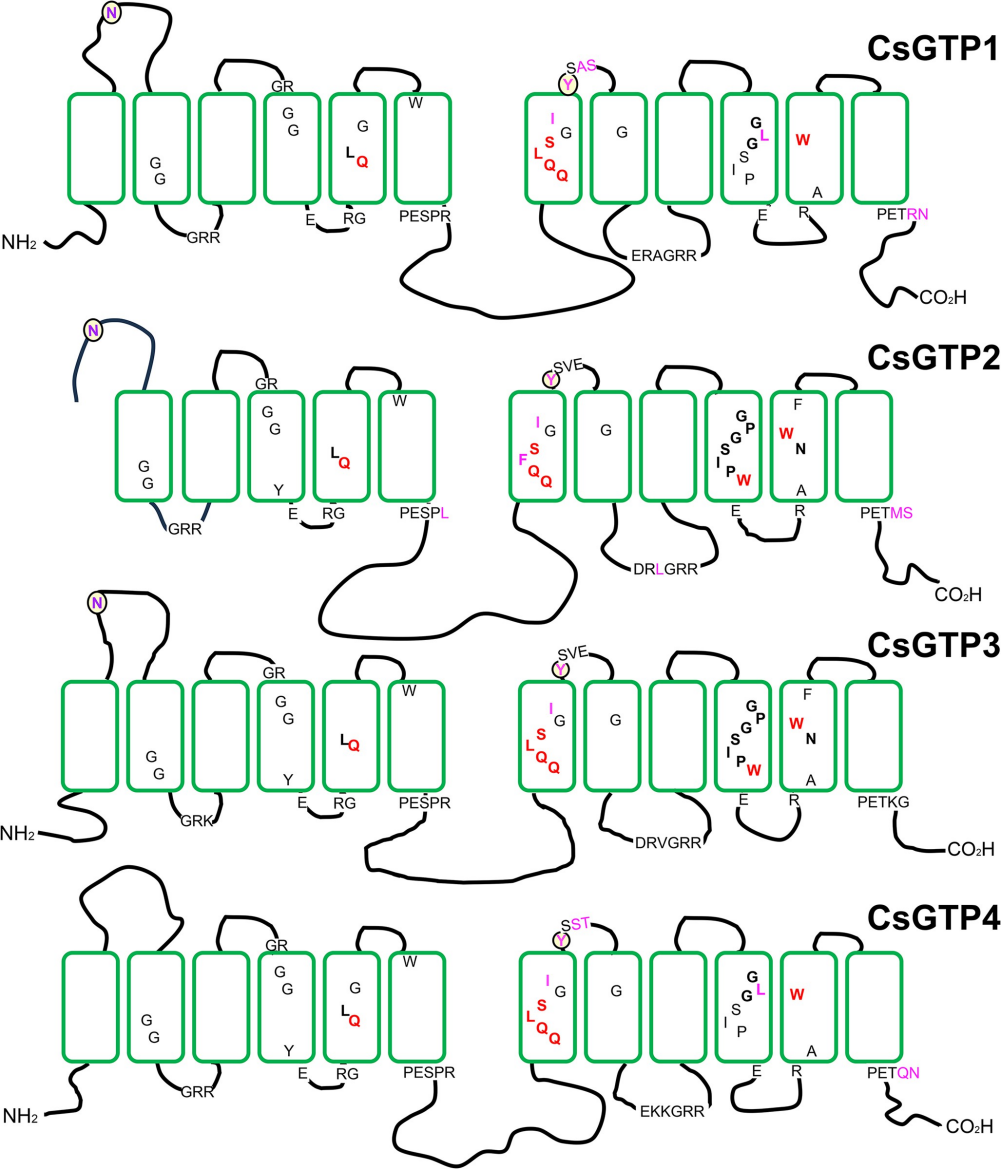
Schematic topology of *C*. *sinensis* glucose transporter (CsGTP) subtypes. Subtypes possess 12 TMs (boxes) except CsGTP2, a large cytoplasmic loop between TM6 and TM7, and a long cytoplasmic C-terminus. Highly conserved functional residues are indicated by letters, of which the red letters are the glucose binding sites.

CsGTP1, 3, and 4 were predicted to have 12 transmembrane domains (TMs) and 11 loops between the TMs, whereas CsGTP2 was predicted to have 11 TMs (Figs 1 and 2). As inferred from the *CsGTP2* cDNA sequence, the polypeptide sequence of the ORF encountered three stops in 5′- region, resulting in a premature stretch without the first methionine at the N-terminus. Nevertheless, the CsGTP2 premature polypeptide retained 11 TMs, and most of the conserved functional motifs and residues of glucose transporter 1 (GLUT1) (S2 Table).

### Secondary structural features of CsGTPs

The CsGTP polypeptides contained 12 TMs, 11 loops, and cytoplasmic N- and C-termini. Functional motifs and key residues identified in vertebrate and invertebrate glucose transporters were highly conserved across all four CsGTPs (Figs 1 and 2, and S2 Table). The conserved motifs in CsGTPs were the PESPR motif in loop6, the PETKG/xx motif in the C-terminus, GRR in loop2 and loop8, WP in TM6, and GP/LGxxP in TM10. For functional conservation, the N-glycosylation site appeared in loop1 of CsGTP1, 2, and 3. The Ex_6_R motif is highly conserved in loop4 and 10 of all CsGTPs and its activity is required for conformational changes and glucose transport [24]. The Q residue in the HQLx_4_G motif of TM5 plays a critical role in transporting glucose to the inward gate [25–27], and is conserved in CsGTPs. In TM7, the QQLS and INA motifs select glucose and move into the extracellular vestibule, and the Y residue closes the vestibule [25,28–30]. In CsGTPs, the QQ, IN, and Y residues are conserved. The aspartate or glutamate residues of the D/ExxGRR motif in loop8 are crucial for GTP function [24] and they are conserved in CsGTPs (Fig 2). Two W residues, located in TM10 and 11 respectively, bind glucose and facilitate its inward movement. These residues are conserved in CsGTP2 and 3, but not in TM10 of CsGTP1 and 4. Additionally, one asparagine residue is highly conserved in TM7 of all CsGTPs and SGTPs and aids in moving glucose to the inner pocket of the transporter [25,31]. Compared to *S*. *mansoni* GTPs, CsGTP1 and 4 are similar to SGTP1 and 4 and are considered to belong to the fluke-specific group, whereas CsGTP2 and 3 are similar to SGTP2 and 3 and are considered to belong to the GLUT-like group.

Compared to human GLUT1, eight residues were found to be conserved only in trematode GTPs: A in loop2 (V in GLUT1), G in TM3 (S in GLUT1), K in loop6 (R in GLUT1), P in TM12 (I in GLUT1), and L in loop6, TM9, and TM11 (Y, H, A, and T in GLUT1). Among them, four residues shared similar properties with those of GLUT1 (V→A, R→K, I→P, and A→L), whereas the remaining four had different properties from those of GLUT1 (S→G, Y→L, H→L, and T→L) (Fig 1).

### CsGTPs grouped into two subgroups

**Fig 3:**
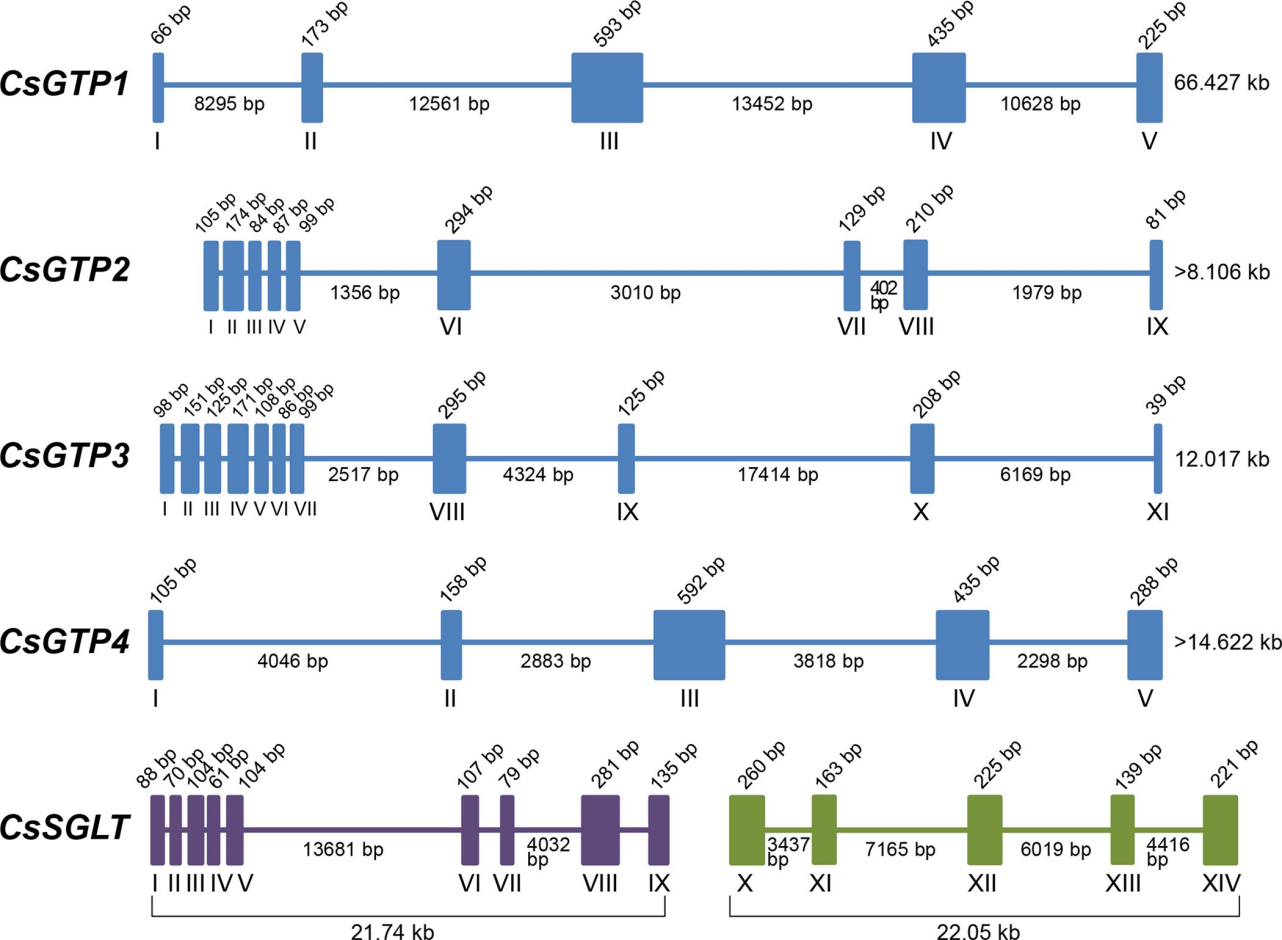
Genomic organization of *C*. *sinensis* glucose transporters (CsGTPs). Solid bars represent exons and the lines between them represent introns. The location of each GTP gene in the *C*. *sinensis* genome is as follows: *CsGTP1* in scaffold 267, DF143094; *CsGTP2* in scaffold 377, DF143204; *CsGTP3* in scaffold 1341, DF144168; *CsGTP4* in scaffold 1341, DF144168. The *CsSGLT* gene is located in two different scaffolds. Exons I–IX are in scaffold 1097, DF143924 (purple) and exons X–XIV are in scaffold 1651, DF144478 (green).

In multiple alignments, CsGTP2 and CsGPT3 exhibited high homology with SGTP2, SGTP3, and GLUT1, whereas CsGTP1 and CsGTP4 were close to SGTP1 and SGTP4 and shared several other conserved residues with SGTP2 and SGTP3 (Fig 1). Genomic organization analysis revealed the existence of two subgroups among the four CsGTPs. CsGTP2 and CsGTP3 were exon-rich, each containing more than 9 exons, whereas CsGTP1 and CsGTP4 contained 5 exons each (Fig 3).

**Fig 4:**
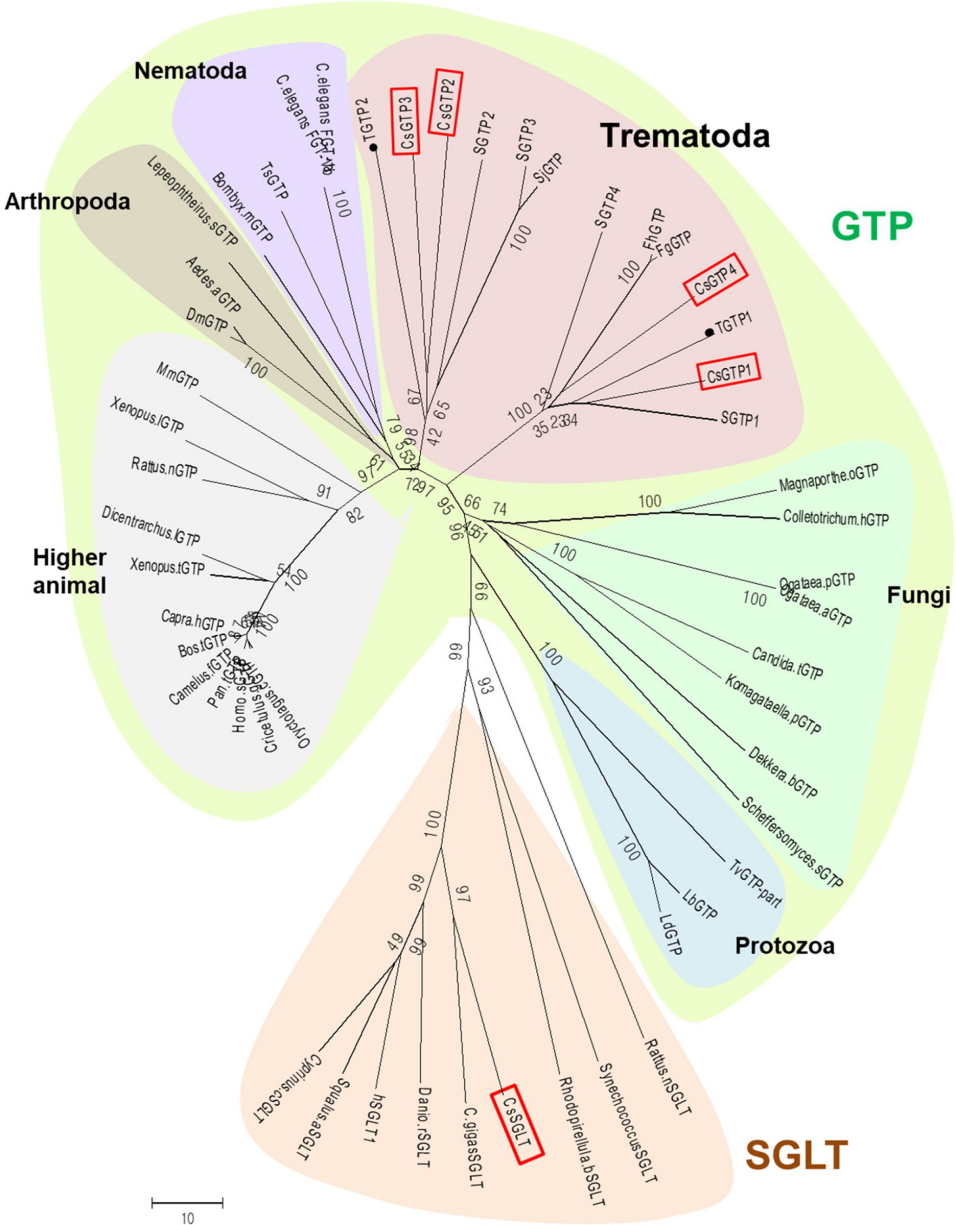
Phylogenetic tree of glucose transporters (GTPs) and sodium glucose co-transporters (SGLTs). CsGTP, *C*. *sinensis* GTP; SGTP, *S*. *mansoni* GTP; SjGTP, *S*. *japonicum* GTP; FhGTP, *Fasciola hepatica* GTP; FgGTP, *F*. *gigantica* GTP; TGTP, *Taenia solium* GTP; CsSGLT, *C*. *sinensis* SGLT. Red boxes, *C*. *sinensis* transporters. TGTP1 and 2 (filled circles) are the GTPs of Cestoda, not Trematoda. This tree was generated using the neighbor-joining method in MEGA5.

A similar organization has also been reported for *S*. *mansoni*, where SGTP2 and SGTP3 exhibit a complex exon arrangement and are closely related to the human GLUT1 family. Additionally, SGTP1 and SGTP4 exhibit parasite specificity and a simple exon arrangement that performs most of the glucose transport functions [29,32]. The genomic organization patterns of CsGTPs were similar to those of SGTPs. Moreover, this genome organization-based classification was supported by the phylogenetic tree, in which CsGTP2 and CsGTP3 were grouped into one subgroup together with trematode GTP2 and GTP3, while CsGTP1 and CsGTP4 were grouped into another subgroup together with trematode GTP1 and GTP4 (Fig 4). This suggests that CsGTP2 and CsGTP3 evolved earlier and are closer to the class I glucose transporter family in higher animals. In contrast, CsGTP1 and CsGTP4 evolved relatively later into a platyhelminth glucose transporter subgroup [32].

### Genetic organization of the sodium glucose co-transporter in *C*. *sinensis* (CsSGLT)

**Fig 5:**
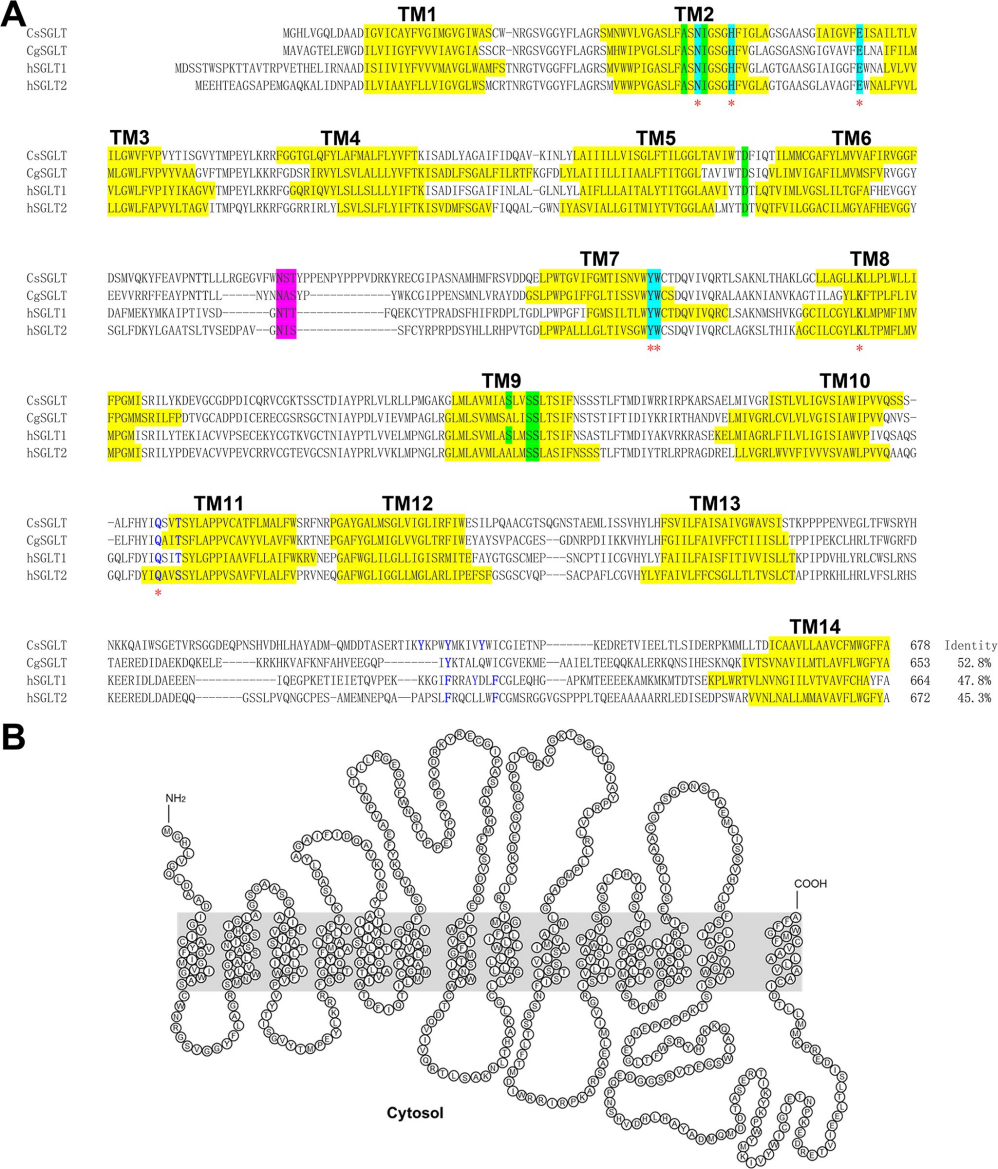
Secondary structure and membrane topology of *C*. *sinensis* sodium glucose co-transporter (CsSGLT) polypeptide. (A) Multiple alignment of CsSGLT with SGLTs from other species. Cg, *Crassostrea gigas*; hSGLT, human SGLT. Yellow and pink backgrounds represent the transmembrane (TM) domain and N-glycosylation sites, respectively. Cyan and green backgrounds represent sodium ion 1 (Na1)- and sodium ion 2 (Na2)-binding residues, respectively. Red asterisk indicates glucose binding residue. (B) Putative membrane architecture of CsSGLT with 14 TM domains, and intra- and extracellular loops.

When reassembled into the master cDNA clone (CSA17668), all 77 ESTs of CL25Contig3 were aligned and tiled onto a template cDNA sequence (S2 Fig). Following a BLAST search, the putative polypeptide of the master clone was found to be premature at the N-terminus. The cDNA stretch obtained through 5′-RACE was elongated by 128 aa, including the first methionine at the N-terminus, and completed with the CsSGLT polypeptide of 678 aa (Figs 5 and S11). CsSGLT was predicted to contain 14 TM domains in the cell membrane, with the N- and C-termini facing outside the cell membrane (Fig 5). It was grouped into one cluster consisting of diverse clades in the phylogenetic tree of GTPs (Fig 4). The full-length sequence of CsSGLT was registered in NCBI under accession number OR678202.

Using a BLASTP search of the entire CsSGLT polypeptide in NCBI, the hypothetical protein, sodium/glucose co-transporter 4 (protein ID: GAA34320), was found in the *C*. *sinensis* genome database, encompassing a posterior region of 335 aa (exons X to XIV). This represented a short, partial CsSGLT, lacking the anterior half of 343 aa. The cDNA sequence of the anterior half was subjected to a BLASTN search using the following options: option 1 –[Database]: others (whole-genome shotgun contigs); option 2 –[Limit by]: organism (*Clonorchis sinensis*–taxid: 79923). Thus, nine anterior exons were discovered on another DNA scaffold. Taken together, the *CsSGLT* cDNA consisted of 14 exons assembled by cis- and trans-splicing from two different DNA scaffolds of the *C*. *sinensis* genome. Exons I–IX resided in DNA scaffold #1097 (GenBank: DF143924) spanning 21.74 kb, and exons X–XIV came from scaffold #1651 (GenBank: DF144478) spanning 22.05 kb (Fig 3). A hypothetical protein (sodium/glucose co-transporter 1; protein ID: GAA41134) included exons V–IX. Exons I–IV of CsSGLT, which are small and close to each other, were identified for the first time in this study. Compared with the exon map of human SGLTs (hSGLTs), CsSGLT showed unique features with some similarities. All hSGLTs, like CsSGLT, were exon-complexed and consisted of 15 exons spanning 8–72 kb. However, the genomic organization pattern of CsSGLT differed from that of hSGLTs. The exon-rich region was located in the middle (hSGLT1 and 4) or posterior (hSGLT2, 5, and 6) of hSGLTs [33], whereas it was located in the anterior part of CsSGLT (Fig 3).

### CsSGLT conserved the signatures of the SGLT family

The CsSGLT polypeptide shared 52.8% sequence identity with the SGLT of *Crassostrea gigas* (GenBank: AAT44356.1) and 47.8 and 45.3% sequence identity with hSGLT1 and hSGLT2, respectively (Fig 5). At the secondary structural level, CsSGLT has conserved consensus regions in loop1 and TM2 [34] (S3 Table). An N-glycosylation is conserved in the extracellular-facing loop6 [35]. The Na^+^ binding sites (Na1 and Na2) are highly conserved in CsSGLT [35]. The residues that interacted with Na1 were N and H in TM2, E in TM3, and Y and W in TM7 (S3 Table). Meanwhile, the residues involved in glucose binding were K in TM8 and Q in TM11 [36,37]. The Na2 binding site, consisting of A and I in TM2, D in loop5, and three Ss in TM9 [38], is also highly conserved in CsSGLT. The sugar moiety of phlorizin interacts with Q457 and T460 of TM11 in hSGLT1, which are highly conserved in CsSGLT (S3 Table). Based on these secondary structural similarities, the putative polypeptide was annotated as CsSGLT (Fig 5).

### Distinctive tissue localization of CsGTPs and CsSGLT

**Fig 6:**
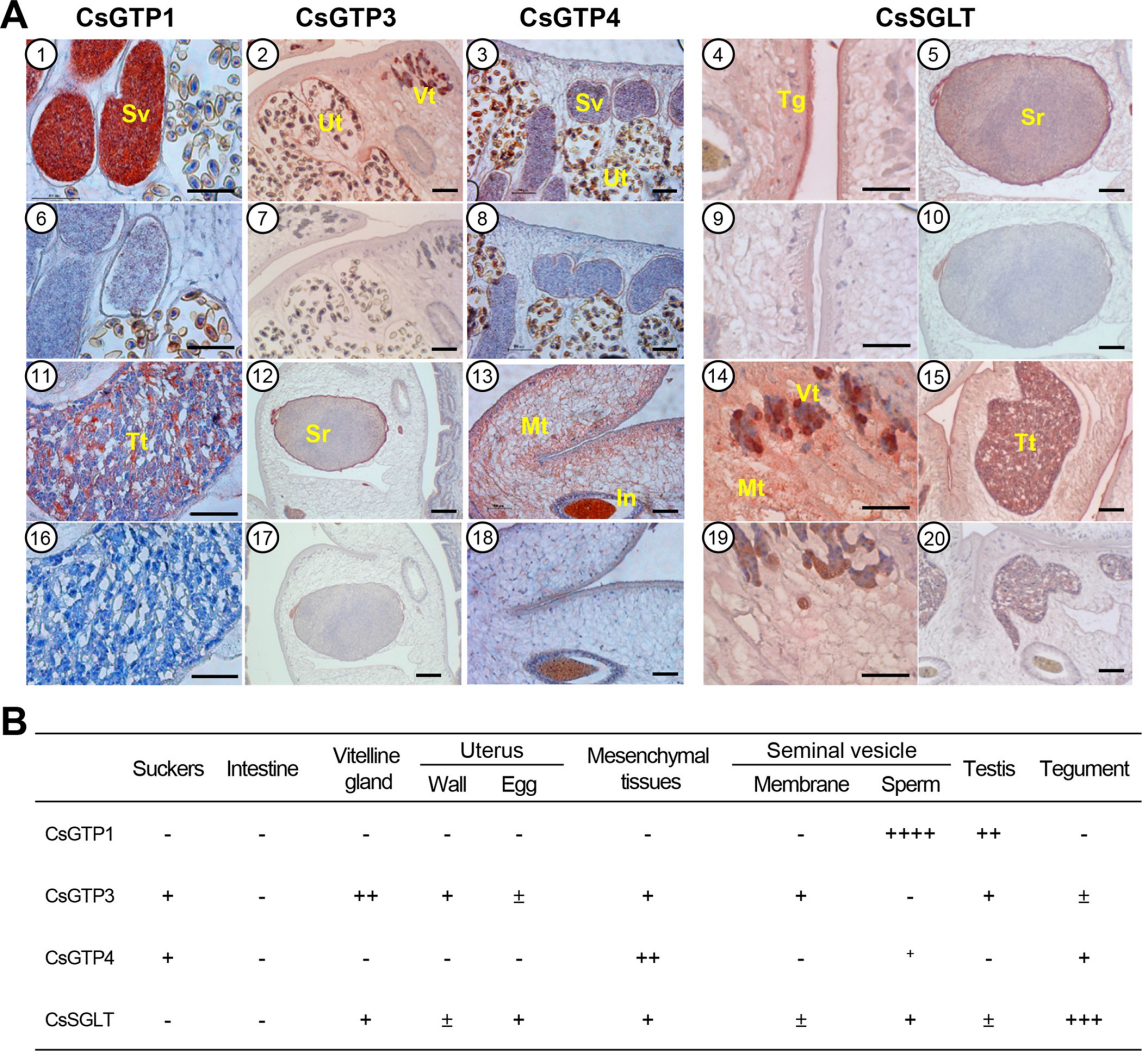
Tissue distribution of *C*. *sinensis* glucose transporters (CsGTPs) and sodium glucose co-transporter (CsSGLT) in adult *C*. *sinensis*. (A) Representative micrograph of immunohistochemical staining for glucose transporters. Tissues of 1–5 and 11–15 were treated with respective mouse immune serum of CsGTP1, CsGTP3, CsGTP4, or CsSGLT. Tissues of 6–10 and 16–20 were treated with normal mouse serum. In, intestine; Mt, mesenchymal tissue; Sr, seminal receptacle; Sv, seminal vesicle; Tg, tegument; Tt, testis; Vt, vitelline gland; Ut, uterus. Scale bar indicates 100 μm. (B) Relative intensity of immunohistochemical staining. Color intensity was arbitrarily scaled with ‘++’ for CsGTP4 in mesenchymal tissue and ‘++++’ for CsGTP1 in seminal vesicle.

Recombinant chimeric antigen proteins (rCsGTP1, rCsGTP3, rCsGTP4, and rCsSGLT) were produced bacterially and purified using Ni-NTA column chromatography. These chimeric proteins were used to immunize BALB/c mice and produce antigen-specific antibodies in the serum (S5 Fig). Immunohistochemical staining (Fig 6) showed that CsGTP1 was localized to the sperm and testes of *C*. *sinensis* adults. It was especially abundant in mature sperm in the seminal vesicle. Therefore, CsGTP1 was designated as the sperm GTP of *C*. *sinensis*. CsGTP3 was distributed in the mesenchymal tissue, wall of the seminal receptacle, vas deferens, testicular sperm, tegument, and sucker. CsGTP4 was distributed in the mesenchymal tissue, seminal vesicle sperm, and tegument. CsSGLT was densely distributed in the tegument and, to a moderate extent, in the mesenchymal tissue, vitelline gland, seminal vesicle sperm, and intrauterine eggs.

### Developmental expression of glucose transporters

**Fig 7:**
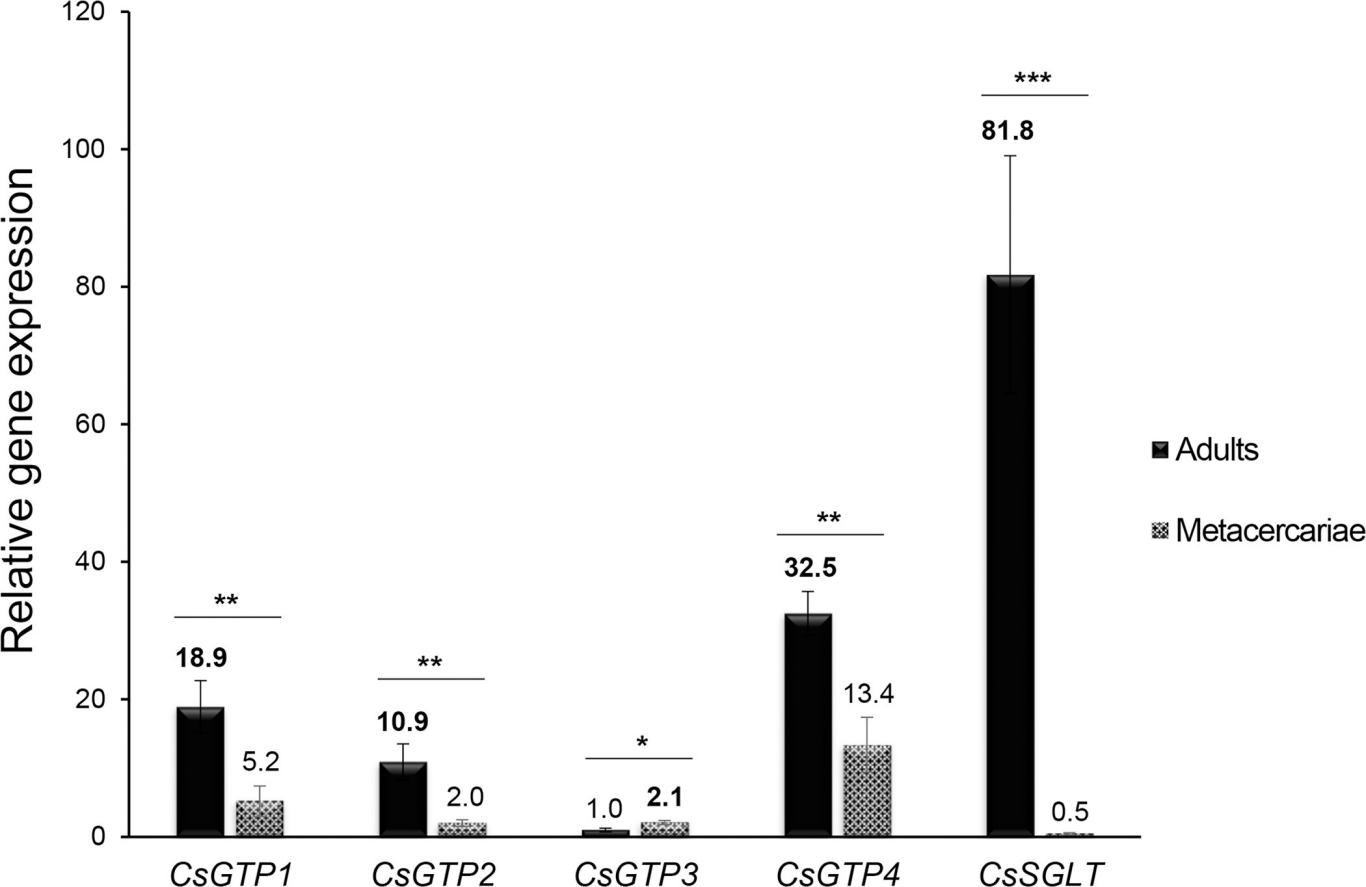
Relative mRNA levels of *C*. *sinensis* glucose transporters. The relative levels of *CsGTP1–4* and *CsSGLT* mRNA in adults and metacercariae were quantified using RT-qPCR and 2^−ΔΔCT^ equation and adult *CsGTP3* as a control. *p < 0.05, **p < 0.01, and ***p < 0.001.

The relative mRNA transcript levels of five glucose transporters in the developmental stages of *C*. *sinensis* were quantified using reverse-transcription quantitative PCR (RT-qPCR) (Fig 7 and S1 Data). CsGTP1, CsGTP2, and CsGTP4 were expressed at 3.6-, 5.5-, and 2.4-fold higher levels in adults than in metacercariae, respectively. In contrast, CsGTP3 expression was 2.1-fold higher in metacercariae than in adults. CsSGLT was barely expressed in metacercariae and was expressed 163.6 times higher in adults than in metacercariae. In adults, the relative mRNA transcript levels of CsGTP1 and CsGTP4 to CsGTP3 were 18.9 and 32.5, respectively. CsSGLT transcript levels were 81.8-fold higher than those of CsGTP3 and 1.6-fold higher than the total transcript levels of the three CsGTPs. This finding was consistent with that of the glucose transporters in *S*. *mansoni* (SGTPs). Among the four SGTPs, SGTP1 and SGTP4 are parasite-specific [32] and essential for the survival of blood flukes [16].

### Glucose uptake in live *C*. *sinensis* adults and the effects of transporter inhibitors

**Fig 8:**
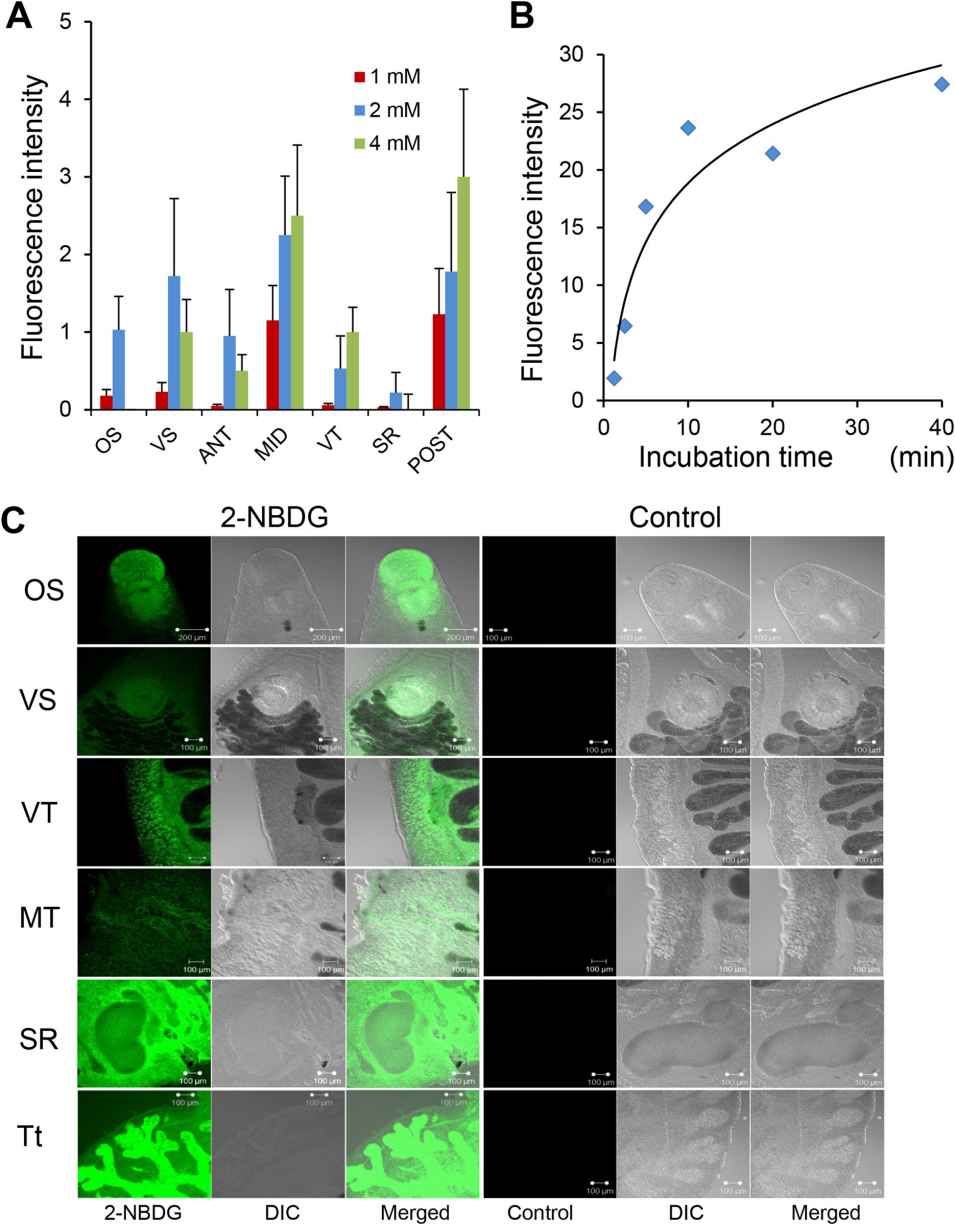
Glucose uptake and distribution in living *C*. *sinensis* adults. (A) 2-*N*-(7-nitrobenz-2-oxa-1,3-diazol-4-yl)amino]-2-deoxy-d-glucose (2-NBDG) absorption in *C*. *sinensis* organs. (B) Time-dependent uptake of 2 mM 2-NBDG. Plotted points are from grouped worm fluorescence intensity data. Three worms were assigned to each group. (C) Distribution of 2-NBDG in live *C*. *sinensis* adults. Flukes were incubated in 1× Locke’s solution containing fluorescent 2-NBDG for 5 min and florescence intensity was measured by confocal microscopy. OS and VS, oral and ventral suckers, respectively; VT, vitelline glands; MT, mesenchymal tissue; SR, seminal receptacle, Tt, testis; ANT, MID, and POST, anterior, middle, and posterior parts of the whole body, respectively.

Adult *C*. *sinensis* absorbed 2-[*N*-(7-nitrobenz-2-oxa-1,3-diazol-4-yl)amino]-2-deoxy-d-glucose (2-NBDG), an exogenous fluorescent glucose analog, in a concentration-dependent manner. The optimal concentration was determined to be 2 mM (Fig 8A and S2 Data). The flukes rapidly absorbed 2-NBDG within 10 min, particularly in the first 5 min. After 10 min, the 2-NBDG absorption was saturated (Fig 8B). In adult *C*. *sinensis*, 2-NBDG was distributed throughout the body in the oral and ventral suckers, pharynx, vitelline follicles, testes, and mesenchymal tissues, with stronger fluorescence in the locomotive and reproductive organs (Fig 8C).

**Fig 9:**
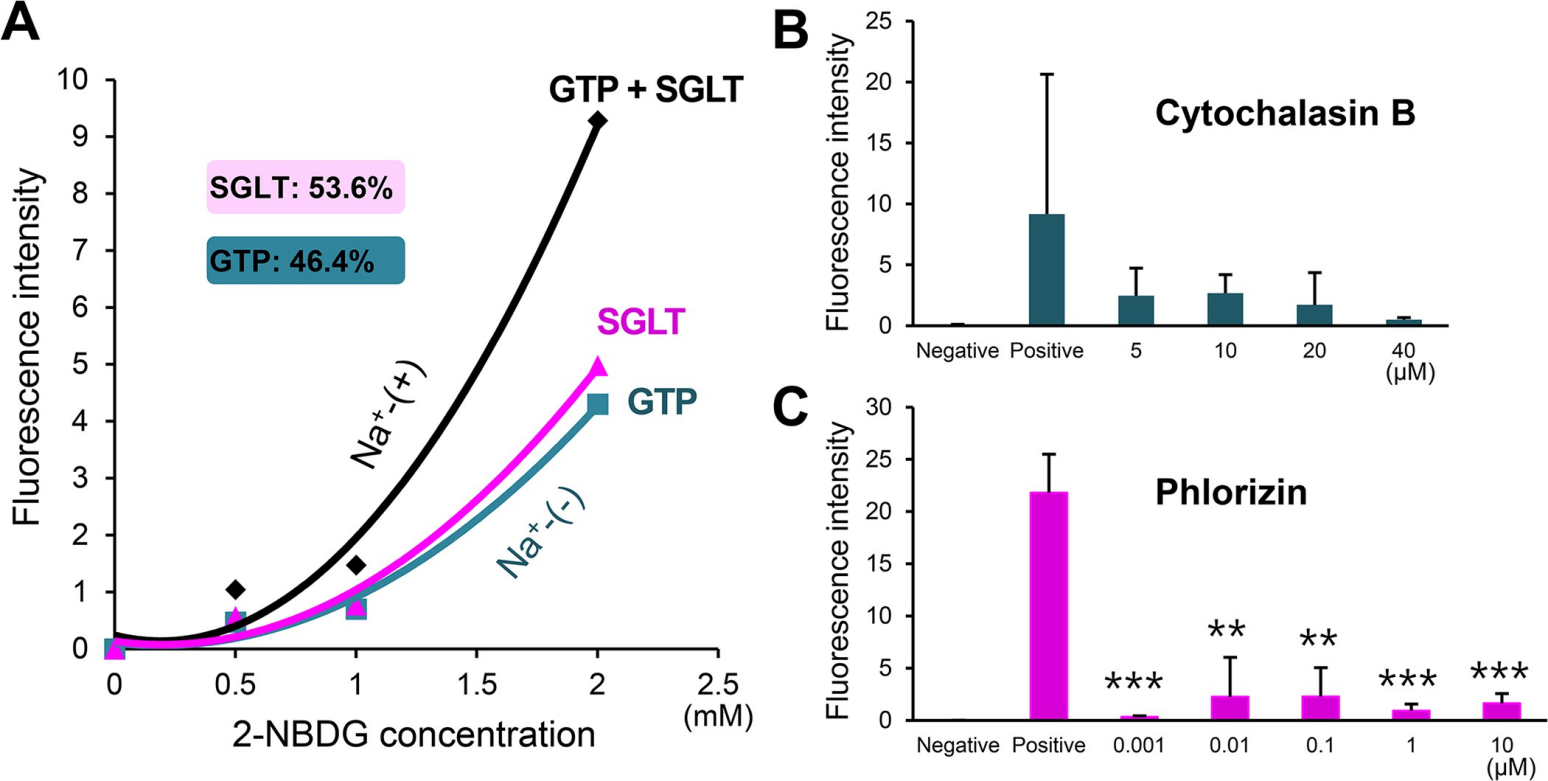
*In vivo* glucose transport via CsGTPs and CsSGLT in adult *C*. *sinensis*. (A) 2-NBDG uptake by total CsGTPs and CsSGLT. Transport by GTPs was measured in Na^+^-depleted glucose medium [Na^+^-(−)]. (B, C) Inhibition of 2-NBDG uptake by a GTP inhibitor (cytochalasin B) and an SGLT inhibitor (phlorizin), respectively. **p < 0.01 and ***p < 0.001 compared to positive control.

For exogenous glucose uptake, CsSGLT plays a more important role (53.6%) compared to CsGTPs (46.4%) (Fig 9A and S3 Data). Cytochalasin B, a GTP inhibitor, effectively inhibited 2-NBDG absorption by 73–95% in a concentration-dependent manner (Fig 9B and S3 Data). Phlorizin, a well-known SGLT inhibitor, showed greater potency, inhibiting more than 95% of 2-NBDG absorption at a concentration of 1 nM (Fig 9C and S3 Data).

## Discussion

Because glucose is an essential nutrient for most organisms, including parasites, starving parasites by cutting off their source of glucose represents a good treatment strategy. Trematodes rely on anaerobic metabolism [39]. Adult schistosomes consume as much glucose as their dry body weight in five hours [40]. Adult *C*. *sinensis*, an anaerobic trematode, exhibits a glucose uptake rate comparable to that of schistosomes and consumes up to 1.13 mg glucose per gram of wet body weight per hour [41]. In trematodes, glucose transporters are present on the tegumental cell membrane and transport large amounts of exogenous glucose. The molecular features and functionality of glucose transporters in *S*. *mansoni* have been previously reported [13,14]. Thus, it was presumed that *C*. *sinensis* possesses transporters that import exogenous glucose and distribute it throughout the body for energy metabolism. In this study, we discovered four glucose transporters and one sodium glucose co-transporter in adult *C*. *sinensis* and reported here their molecular identities, features, functions, and implications.

In the putative topology, all CsGTPs had a large cytoplasmic loop between TM6 and TM7 and a large cytoplasmic tail after TM12. In contrast, CsSGLT has two large extracellular loops between TM6 and TM7, TM8 and TM9, and a large cytoplasmic loop between TM13 and TM14. Producing whole stretches of membrane proteins in recombinant forms using bacterial cells is challenging. To overcome this hurdle, two intracellular and extracellular loop regions of the three CsGTPs and CsSGLT were subcloned and successfully produced as recombinant chimeric proteins in bacterial cells. These four recombinant proteins were antigenic and suitable for subsequent downstream experiments aimed at generating individual glucose transporter-specific immune serum and performing differential immunohistochemical staining for each glucose transporter in adult *C*. *sinensis*.

In the *C*. *sinensis* EST database, contigs encoding CsGTPs and CsSGLT were found to be more abundant in adults than in metacercariae (S1 Table), which was consistent with the developmental level of the gene transcripts in *C*. *sinensis*. The expression levels of CsGTPs and CsSGLT were higher at the adult stage than at the metacercarial stage. Higher expression of glucose transporters in adults accounts for the active uptake of large amounts of glucose to generate energy and metabolic intermediates for various physico-metabolomic requirements such as movement in the host bile duct, reproduction, and evasion of host immune attacks [5].

CsSGLT is predominantly expressed in *C*. *sinensis* adults. During *C*. *sinensis* development, metacercariae are in a resting state within the cyst, requiring minimal energy, as they cannot absorb glucose from outside of this environment. Therefore, CsSGLT is not expressed in the metacercariae [42]. In comparison, adult *C*. *sinensis* is in an active reproductive stage, producing large quantities of eggs and sperm, within the bile ducts of mammalian hosts. Therefore, *C*. *sinensis* adults require large amounts of external nutrients, particularly glucose, as an energy source. CsSGLT is distributed throughout the body surface in adults to facilitate active absorption of glucose from bile, where the glucose concentration is typically low [43]. In *Taenia saginata*, SGLT is abundant on the proglottid tegument surface and on the walls of the uterine branches, suggesting that glucose uptake is an important function in the reproductive organs of adult tapeworms [44]. CsSGLT is mainly found in the tegument and accounts for more than half of the exogenous glucose uptake in *C*. *sinensis* adults. In human bile, Na^+^ ions are present at high molar concentrations [45]. This high Na^+^ environment may help *C*. *sinensis* efficiently uptake exogenous glucose from bile by CsSGLT.

In adult *C*. *sinensis*, CsGTPs are distributed throughout the internal organs and mesenchymal tissues in distinct patterns, suggesting that they have various functions in each organ. As single transporters, CsGTPs are less prominently distributed in the tegument of *C*. *sinensis*. However, in a glucose-rich *in vitro* environment, CsGTP1, 3, and 4 take up exogenous glucose with an efficiency similar to that of CsSGLT [14–16]. After ingestion, glucose moves to the mesenchymal tissues, vitelline glands, and testes, where vitellogenic and spermatogenic cells are actively produced. With increased incubation times, higher 2-NBDG concentrations were observed in the vitelline glands and testes. In *Haploporus lateralis*, a trematode, the vitelline gland produces vitelline cell that provide shell components and nutrients to the embryo [46]. To meet their physiological needs, the vitelline gland takes up glucose through glucose transporters, most likely CsGTP4, orchestrated by CsGTP3 and CsSGLT in *C*. *sinensis* adults [14,16].

In adult *C*. *sinensis*, CsGTP1 is distributed distinctively and at a high density in the sperm of the seminal vesicles and testes. However, CsGTP is sparse in the sperm of the seminal receptacle. Functional analysis revealed no 2-NBDG fluorescence in the seminal receptacles. This suggests that when sperm become more active, more CsGTPs are needed to provide the increased glucose requirements as an energy source. Meanwhile, when sperm are in the resting stage within the seminal receptacle, their glucose requirements decrease and CsGTP expression is minimized. However, this finding warrants future research.

Adult *C*. *sinensis* parasitizes the biliary tract, holding its position using oral and ventral suckers. To inhabit this parasitic habitat, the liver fluke must constantly move its two suckers and the muscle fibers of its mesenchymal tissue. Glucose is required to provide energy for this survival movement. Muscle fibers in the mesenchymal tissue may receive glucose through CsGTP4, and oral and ventral suckers through CsGTP3 and 4.

## Conclusions

Praziquantel has an efficient anthelmintic effect against trematodes such as schistosomes, *C*. *sinensis*, *Paragonimus westermani*, and the tapeworm *Diphyllobothrium latum*. However, owing to their repeated use, praziquantel-resistant strains have emerged [47–50]. Glucose is a prerequisite for the survival of trematodes such as *C*. *sinensis* and *S*. *mansoni* [14]. Functional and inhibitory studies of glucose transporters may pave the way for the development of new anthelmintic drugs. Cytochalasin B and phlorizin interfere with the diffusion of glucose through GTPs. Sodium glucose co-transporters require the hydrolysis of ATP and are sensitive to inhibition by phlorizin, ouabain (a Na^+^/K^+^-ATPase inhibitor), and monensin (a Na^+^ ionophore) [51]. Functional studies of glucose transporters using inhibitors should be conducted in the near future.

## Supporting information

S1 Fig *C*. *sinensis* glucose transporter (CsGTP) expressed sequence tags (ESTs) aligned with the master clone cDNA sequence at the top of each subtype. The percentage of each EST indicates the sequence identity with the master clone. CsGTP3 consists of CL272 and CL3450, CsGTP2 consists of CL1676 and CL3618, CsGTP4 consists of ESTs in CL1983, and CsGTP1 consists of CL353, CL2607 and CSA24824.
(TIF)

S2 Fig Alignment of *C*. *sinensis* sodium glucose co-transporter (CsSGLT) expressed sequence tags (ESTs) in CL25Contig3. EST sequences were identical to those of the master clone cDNA. The full-length cDNA of the master clone was obtained by 5′-RACE from the total cDNA of adult *C*. *sinensis*.
(TIF)

S3 Fig Comparison of four *C*. *sinensis* glucose transporter (CsGTP) polypeptide sequences derived from cDNAs in the present experimental study and putative cDNAs from genomic sequences. The hatched regions represent identical peptide sequences of the experimental and putative cDNAs, whereas the solid regions are different. GAAnnnnn is a putative CsGTP sequence obtained from GenBank (NCBI, NIH, USA).
(TIF)

S4 Fig Chimeric antigenic protein design by selecting B-cell epitopes (top) and hydrophilic regions (bottom). (A) CsGTP1; (B) CsGTP3; (C) CsGTP4; (D) CsSGLT. Pink boxes indicate B-cell epitopes matching hydrophilic regions. The two segments were bridged using the spacer peptide GPGPG, allowing the freedom of the segments.
(TIF)

S5 Fig Recombinant protein production. All recombinant proteins were purified by Ni-NTA column chromatography. Left panel: fractions were electrophoresed on SDS-PAGE gels and stained with Coomassie blue. Right panel: western blotting of the respective chimeric proteins using mouse immune sera. (A) Chimeric CsGTP1. (B) Chimeric CsGTP3. (C) Chimeric CsGTP4. (D) Chimeric CsSGLT. U: uninduced total fraction; I: induced total fraction; CL: clear lysate; PT: pass-through fraction; W: wash; lane 1–6, eluates from the Ni-NTA column; R: recombinant chimeric protein; N: native crude extract of *C*. *sinensis*; C: control.
(TIF)

S6 Fig **Contigs and expressed sequence tags (ESTs) encoding glucose transporters (A) and sodium glucose co-transporter (B) in the *C*. *sinensis* transcriptome database.** The adult ESTs have a blue background, whereas the egg ESTs have an orange background.
(TIF)

S7 Fig Complete cDNA and deduced polypeptide sequence of CsGTP1. (TIF)

S8 Fig Complete cDNA and deduced polypeptide sequence of CsGTP2. (TIF)

S9 Fig Complete cDNA and deduced polypeptide sequence of CsGTP3. (TIF)

S10 Fig Complete cDNA and deduced polypeptide sequence of CsGTP4. (TIF)

S11 Fig Complete cDNA and deduced polypeptide sequence of CsSGLT. Nucleotide sequence obtained by 5′-RACE is underlined.
(TIF)

S1 Table Expressed sequence tags (ESTs) encoding *C*. *sinensis* glucose transporter (CsGTP) and *C*. *sinensis* sodium glucose co-transporter (CsSGLT). (DOCX)

S2 Table Secondary structural features of *C*. *sinensis* glucose transporter (CsGTP) subtypes: conserved residues and functional motifs. (DOCX)

S3 Table Conserved residues and characteristic functional motifs in *C*. *sinensis* sodium glucose co-transporter (CsSGLT). (DOCX)

S1 Data Spreadsheet containing the numerical data for Fig 7. (XLSX)

S2 Data Spreadsheet containing the numerical data for Fig 8. (XLSX)

S3 Data Spreadsheet containing the numerical data for Fig 9. (XLSX)

## References

[1] Kim TI, Yoo WG, Kwak BK, Seok JW, Hong SJ. Tracing of the bile-chemotactic migration of juvenile *Clonorchis sinensis* in rabbits by PET-CT. PLoS Negl Trop Dis. 2011; 5(12):e1414. 22180795 10.1371/journal.pntd.0001414 PMC3236719

[2] Li S, Yoo WG, Song JH, Kim TI, Hong SJ. Bile acids drive chemotaxis of *Clonorchis sinensis* juveniles to the bile duct. PLoS Negl Trop Dis. 2018; 12(10):e0006818. 30273341 10.1371/journal.pntd.0006818 PMC6181427

[3] Dai F, Song JH, Hong YP, Bai X, Sohn WM, Hong SJ. Dopaminergic antagonists inhibit bile chemotaxis of adult *Clonorchis sinensis* and its egg production. PLoS Negl Trop Dis. 2020; 14(3):e0008220. 32226018 10.1371/journal.pntd.0008220 PMC7145267

[4] Srivatanakul P, Sriplung H, Deerasamee S. Epidemiology of liver cancer: an overview. Asian Pac J Cancer Prev. 2004; 5(2):118–25. 15244512

[5] Kang IK, Lee SH, Seo BS. Study on the ^14^C-glucose metabolism by *Clonorchis sinensis*: paper chromatographic analyses in combination with autoradiography. Korean J Parasitol. 1969; 7(3):143–52. 10.3347/kjp.1969.7.3.143 12913527

[6] Tielens AGM. The carbohydrate metabolism of *Fasciola hepatica*, an example of biochemical adaptations in parasitic helminths. Acta Parasitol. 2000; 45(2):59–66.

[7] Bueding E. Carbohydrate metabolism of *Schistosoma mansoni*. J Gen Physiol. 1950; 33(5):475–95. 15422103 10.1085/jgp.33.5.475 PMC2147213

[8] Tetaud E, Barrett MP, Bringaud F, Baltz T. Kinetoplastid glucose transporters. Biochem J. 1997; 325:569–80. doi: 10.1042/bj3250569 9271074 PMC1218597

[9] Azema L, Claustre S, Alric I, Blonski C, Willson M, Perie J, et al. Interaction of substituted hexose analogues with the *Trypanosoma brucei* hexose transporter. Biochem Pharmacol. 2004; 67(3):459–67. 15037198 10.1016/j.bcp.2003.09.005

[10] Barrett MP, Tetaud E, Seyfang A, Bringaud F, Baltz T. Trypanosome glucose transporters. Mol Biochem Parasitol. 1998; 91(1):195–205. doi: 10.1016/s0166-6851(97)00192-8 9574935

[11] Slavic K, Straschil U, Reininger L, Doerig C, Morin C, Tewari R, et al. Life cycle studies of the hexose transporter of *Plasmodium* species and genetic validation of their essentiality. Mol Microbiol. 2010; 75(6):1402–13. 20132450 10.1111/j.1365-2958.2010.07060.x PMC2859251

[12] Rodriguez-Contreras D, Skelly PJ, Landa A, Shoemaker CB, Laclette JP. Molecular and functional characterization and tissue localization of 2 glucose transporter homologues (TGTP1 and TGTP2) from the tapeworm *Taenia solium*. Parasitology. 1998; 117:579–88. 9881383 10.1017/s003118209800345x

[13] Skelly PJ, Kim JW, Cunningham J, Shoemaker CB. Cloning, characterization, and functional expression of cDNAs encoding glucose transporter proteins from the human parasite *Schistosoma mansoni*. J Biol Chem. 1994; 269(6):4247–53. 8307988

[14] Skelly PJ, Shoemaker CB. Rapid appearance and asymmetric distribution of glucose transporter SGTP4 at the apical surface of intramammalian-stage *Schistosoma mansoni*. Proc Natl Acad Sci USA. 1996; 93(8):3642–6. 8622989 10.1073/pnas.93.8.3642 PMC39664

[15] Jiang J, Skelly PJ, Shoemaker CB, Caulfield JP. *Schistosoma mansoni*: the glucose transport protein SGTP4 is present in tegumental multilamellar bodies, discoid bodies, and the surface lipid bilayers. Exp Parasitol. 1996; 82(2):201–10. 8617347 10.1006/expr.1996.0025

[16] Krautz-Peterson G, Simoes M, Faghiri Z, Ndegwa D, Oliveira G, Shoemaker CB, et al. Suppressing glucose transporter gene expression in schistosomes impairs parasite feeding and decreases survival in the mammalian host. PLoS Pathog. 2010; 6(6):e1000932. doi: 10.1371/journal.ppat.1000932 20532163 PMC2880588

[17] Feistel T, Hodson CA, Peyton DH, Landfear SM. An expression system to screen for inhibitors of parasite glucose transporters. Mol Biochem Parasitol. 2008; 162(1):71–6. doi: 10.1016/j.molbiopara.2008.07.005 18708094 PMC2771778

[18] Kim DW, Yoo WG, Lee S, Lee MR, Kim YJ, Cho SH, et al. ClonorESTdb: a comprehensive database for *Clonorchis sinensis* EST sequences. BMC Res Notes. 2014; 7:388. 24957044 10.1186/1756-0500-7-388 PMC4094540

[19] Lu Y, Yoo WG, Dai F, Lee JY, Pak JH, Sohn WM, et al. Characterization of a novel organic solute transporter homologue from *Clonorchis sinensis*. PLoS Negl Trop Dis. 2018; 12(4):e0006459. 29702646 10.1371/journal.pntd.0006459 PMC5942847

[20] Krogh A, Larsson B, von Heijne G, Sonnhammer EL. Predicting transmembrane protein topology with a hidden Markov model: application to complete genomes. J Mol Biol. 2001; 305(3):567–80. doi: 10.1006/jmbi.2000.4315 11152613

[21] Dai F, Yoo WG, Lu Y, Song JH, Lee JY, Byun Y, et al. Sodium-bile acid co-transporter is crucial for survival of a carcinogenic liver fluke *Clonorchis sinensis* in the bile. PLoS Negl Trop Dis. 2020; 14(12):e0008952. 33284789 10.1371/journal.pntd.0008952 PMC7746286

[22] Hong SJ, Seong KY, Sohn WM, Song KY. Molecular cloning and immunological characterization of phosphoglycerate kinase from *Clonorchis sinensis*. Mol Biochem Parasitol. 2000; 108(2):207–16. 10838223 10.1016/s0166-6851(00)00220-6

[23] Livak KJ, Schmittgen TD. Analysis of relative gene expression data using real-time quantitative PCR and the 2^-ΔΔCT^ method. Methods. 2001; 25(4):402–8. 11846609 10.1006/meth.2001.1262

[24] Schurmann A, Doege H, Ohnimus H, Monser V, Buchs A, Joost HG. Role of conserved arginine and glutamate residues on the cytosolic surface of glucose transporters for transporter function. Biochemistry. 1997; 36(42):12897–902. doi: 10.1021/bi971173c 9335548

[25] Park MS. Molecular dynamics simulations of the human glucose transporter GLUT1. PloS one. 2015; 10(4):e0125361. doi: 10.1371/journal.pone.0125361 25919356 PMC4412407

[26] Olsowski A, Monden I, Krause G, Keller K. Cysteine scanning mutagenesis of helices 2 and 7 in GLUT1 identifies an exofacial cleft in both transmembrane segments. Biochemistry. 2000; 39(10):2469–74. doi: 10.1021/bi992160x 10704196

[27] Mueckler M, Weng W, Kruse M. Glutamine 161 of Glut1 glucose transporter is critical for transport activity and exofacial ligand binding. J Biol Chem. 1994; 269(32):20533–8. 8051152

[28] Inukai K, Asano T, Katagiri H, Anai M, Funaki M, Ishihara H, et al. Replacement of both tryptophan residues at 388 and 412 completely abolished cytochalasin B photolabelling of the GLUT1 glucose transporter. Biochem J. 1994; 302:355–61. doi: 10.1042/bj3020355 8092986 PMC1137236

[29] Wandel S, Schurmann A, Becker W, Summers SA, Shanahan MF, Joost HG. Substitution of conserved tyrosine residues in helix 4 (Y143) and 7 (Y293) affects the activity, but not IAPS-forskolin binding, of the glucose transporter GLUT4. FEBS Lett. 1994; 348(2):114–8. doi: 10.1016/0014-5793(94)00558-3 8034025

[30] Mori H, Hashiramoto M, Clark AE, Yang J, Muraoka A, Tamori Y, et al. Substitution of tyrosine 293 of GLUT1 locks the transporter into an outward facing conformation. J Biol Chem. 1994; 269(15):11578–83. 8157690

[31] Hruz PW, Mueckler MM. Cysteine-scanning mutagenesis of transmembrane segment 11 of the GLUT1 facilitative glucose transporter. Biochemistry. 2000; 39(31):9367–72. doi: 10.1021/bi000821g 10924131

[32] Cabezas-Cruz A, Valdes JJ, Lancelot J, Pierce RJ. Fast evolutionary rates associated with functional loss in class I glucose transporters of *Schistosoma mansoni*. BMC genomics. 2015; 16:980. 26584526 10.1186/s12864-015-2144-6 PMC4653847

[33] Wright EM, Loo DD, Hirayama BA. Biology of human sodium glucose transporters. Physiol Rev. 2011; 91(2):733–94. doi: 10.1152/physrev.00055.2009 21527736

[34] Wright EM, Loo DD, Hirayama BA, Turk E. Surprising versatility of Na^+^-glucose cotransporters: SLC5. Physiology (Bethesda). 2004; 19:370–6. 15546855 10.1152/physiol.00026.2004

[35] Wright EM, Turk E. The sodium/glucose cotransport family SLC5. Pflugers Arch. 2004; 447(5):510–8. doi: 10.1007/s00424-003-1063-6 12748858

[36] Tyagi NK, Kumar A, Goyal P, Pandey D, Siess W, Kinne RK. D-Glucose-recognition and phlorizin-binding sites in human sodium/D-glucose cotransporter 1 (hSGLT1): a tryptophan scanning study. Biochemistry. 2007; 46(47):13616–28. doi: 10.1021/bi701193x 17983207

[37] Diez-Sampedro A, Barcelona S. Sugar binding residue affects apparent Na^+^ affinity and transport stoichiometry in mouse sodium/glucose cotransporter type 3B. J Biol Chem. 2011; 286(10):7975–82. 21187287 10.1074/jbc.M110.187880 PMC3048684

[38] Loo DD, Jiang X, Gorraitz E, Hirayama BA, Wright EM. Functional identification and characterization of sodium binding sites in Na symporters. Proc Natl Acad Sci USA. 2013; 110(47):E4557–66. doi: 10.1073/pnas.1319218110 24191006 PMC3839715

[39] van Grinsven KW, van Hellemond JJ, Tielens AG. Acetate:succinate CoA-transferase in the anaerobic mitochondria of *Fasciola hepatica*. Mol Biochem Parasitol. 2009; 164(1):74–9. 19103231 10.1016/j.molbiopara.2008.11.008

[40] Githui EK, Damian RT, Aman RA. *Schistosoma mansoni*: biochemical characterization of lactate transporters or similar proteins. Exp Parasitol. 2006; 114(3):180–8. 16682030 10.1016/j.exppara.2006.03.007

[41] Hahn SJ, Hahn HJ, Seo BS. The uptake of C^14^ glucose by *Clonorchis sinensis*. Kor J Int Med. 1961; 4(4):281–5.

[42] Yoo WG, Kim DW, Ju JW, Cho PY, Kim TI, Cho SH, et al. Developmental transcriptomic features of the carcinogenic liver fluke, *Clonorchis sinensis*. PLoS Negl Trop Dis. 2011; 5(6):e1208. 21738807 10.1371/journal.pntd.0001208 PMC3125140

[43] Guzelian P, Boyer JL. Glucose reabsorption from bile. Evidence for a biliohepatic circulation. J Clin Invest. 1974; 53(2):526–35. doi: 10.1172/JCI107586 11344566 PMC301495

[44] Cornford EM, Cornford ME, Wright EM, Bruckner DA, Sampogna S, Hirayama BA. Human cerebral cysticercosis: immunolocalization of a sodium-dependent glucose cotransporter (SGLT) in larval and adult tapeworms. J Parasitol. 2001; 87(3):510–21. doi: 10.1645/0022-3395(2001)087[0510:HCCIOA]2.0.CO;2 11426712

[45] Hofmann AF. Bile composition. In: Johnson LR, editor. Encyclopedia of Gastroenterology: Academic Press; 2003. p. 176–84.

[46] Sampour M. The study of vitelline gland of *Haploporus lateralis* (Digenea: Trematoda). Pak J Biol Sci. 2008; 11(1):113–7. 18819603 10.3923/pjbs.2008.113.117

[47] Ismail M, Botros S, Metwally A, William S, Farghally A, Tao LF, et al. Resistance to praziquantel: direct evidence from *Schistosoma mansoni* isolated from Egyptian villagers. Am J Trop Med Hyg. 1999; 60(6):932–5. 10403323 10.4269/ajtmh.1999.60.932

[48] Danso-Appiah A, De Vlas SJ. Interpreting low praziquantel cure rates of *Schistosoma mansoni* infections in Senegal. Trends Parasitol. 2002; 18(3):125–9. 11854090 10.1016/s1471-4922(01)02209-7

[49] William S, Botros S. Validation of sensitivity to praziquantel using *Schistosoma mansoni* worm muscle tension and Ca^2+^-uptake as possible in vitro correlates to in vivo ED_50_ determination. Int J Parasitol. 2004; 34(8):971–7. 15217736 10.1016/j.ijpara.2004.04.005

[50] Botros S, Sayed H, Amer N, El-Ghannam M, Bennett JL, Day TA. Current status of sensitivity to praziquantel in a focus of potential drug resistance in Egypt. Int J Parasitol. 2005; 35(7):787–91. doi: 10.1016/j.ijpara.2005.02.005 15925597

[51] Silverman M. Glucose transport in the kidney. Biochim Biophys Acta. 1976; 457(3–4):303–51. doi: 10.1016/0304-4157(76)90003-4 136997

