# Supplementary figures and images for: Glucose transporters and sodium glucose co-transporters cooperatively import glucose into energy-demanding organs in carcinogenic liver fluke Clonorchis sinensis
Source: PLoS Negl Trop Dis. 2024 Jul 5;18(7):e0012315. doi: 10.1371/journal.pntd.0012315 (PMC11253919; doi:10.1371/journal.pntd.0012315)

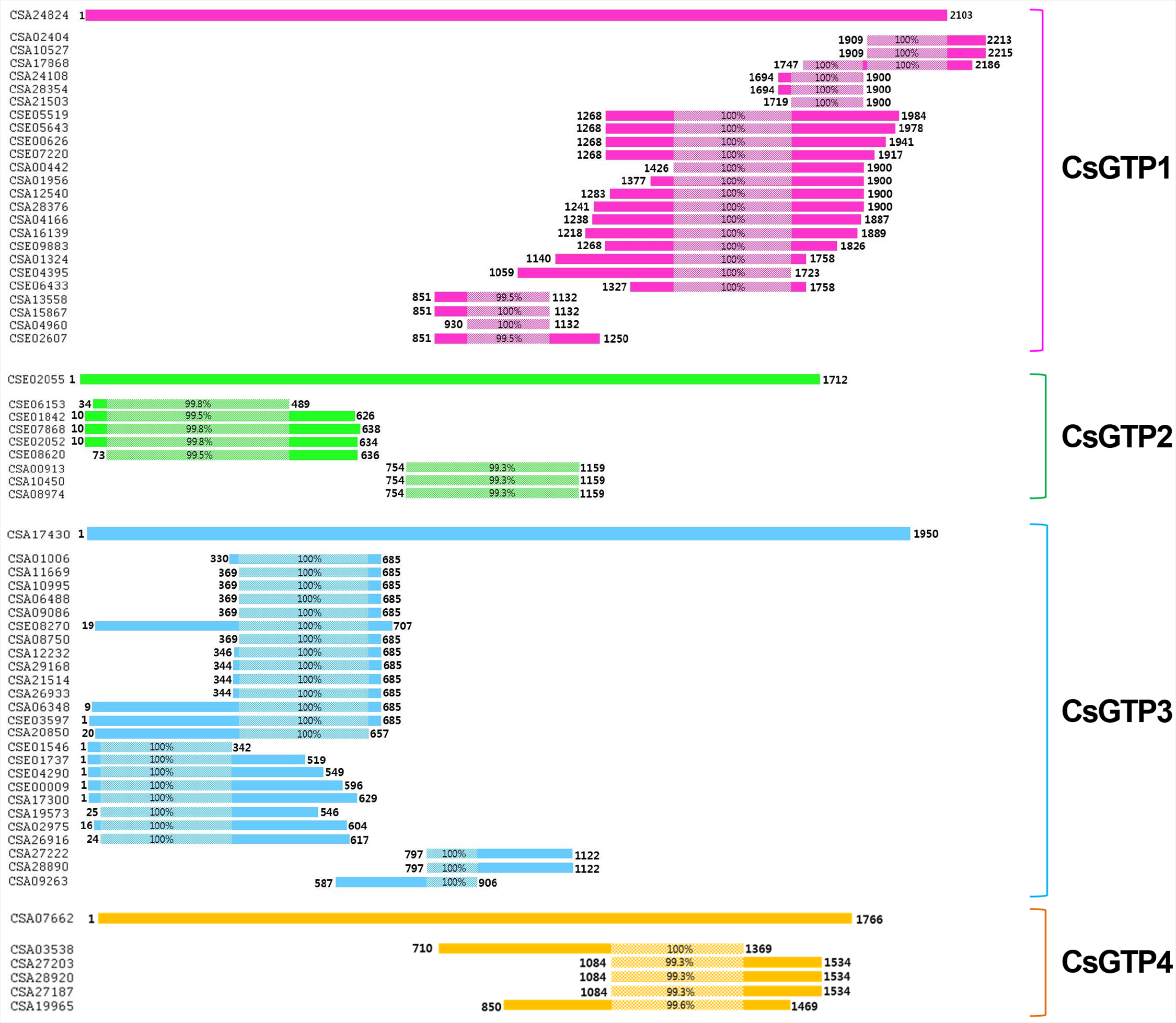

Supplement: S1 Fig — The percentage of each EST indicates the sequence identity with the master clone. CsGTP3 consists of CL272 and CL3450, CsGTP2 consists of CL1676 and CL3618, CsGTP4 consists of ESTs in CL1983, and CsGTP1 consists of CL353, CL2607 and CSA24824. (TIF) [file pntd.0012315.s001.tif]

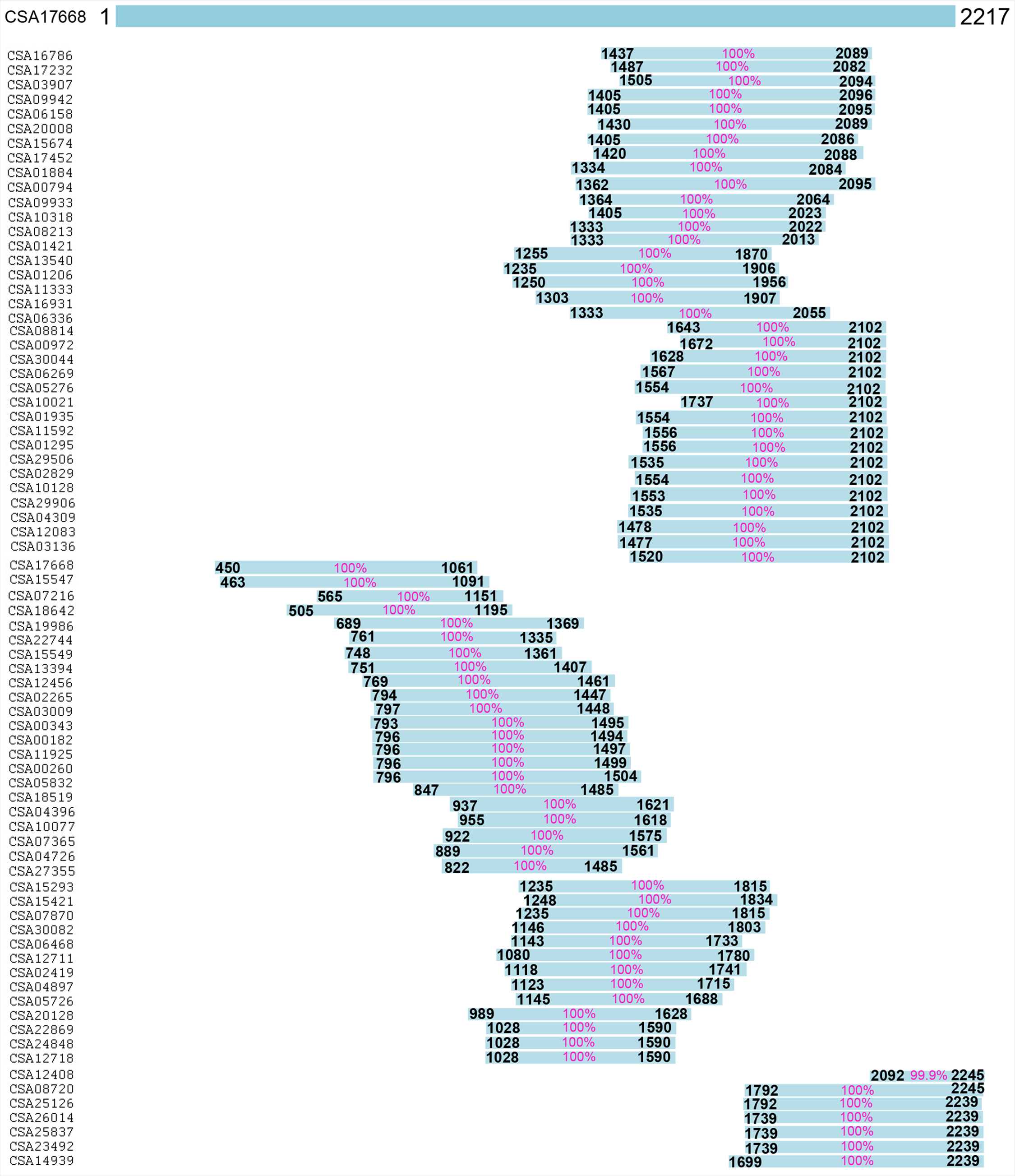

Supplement: S2 Fig — EST sequences were identical to those of the master clone cDNA. The full-length cDNA of the master clone was obtained by 5′-RACE from the total cDNA of adult C. sinensis. (TIF) [file pntd.0012315.s002.tif]

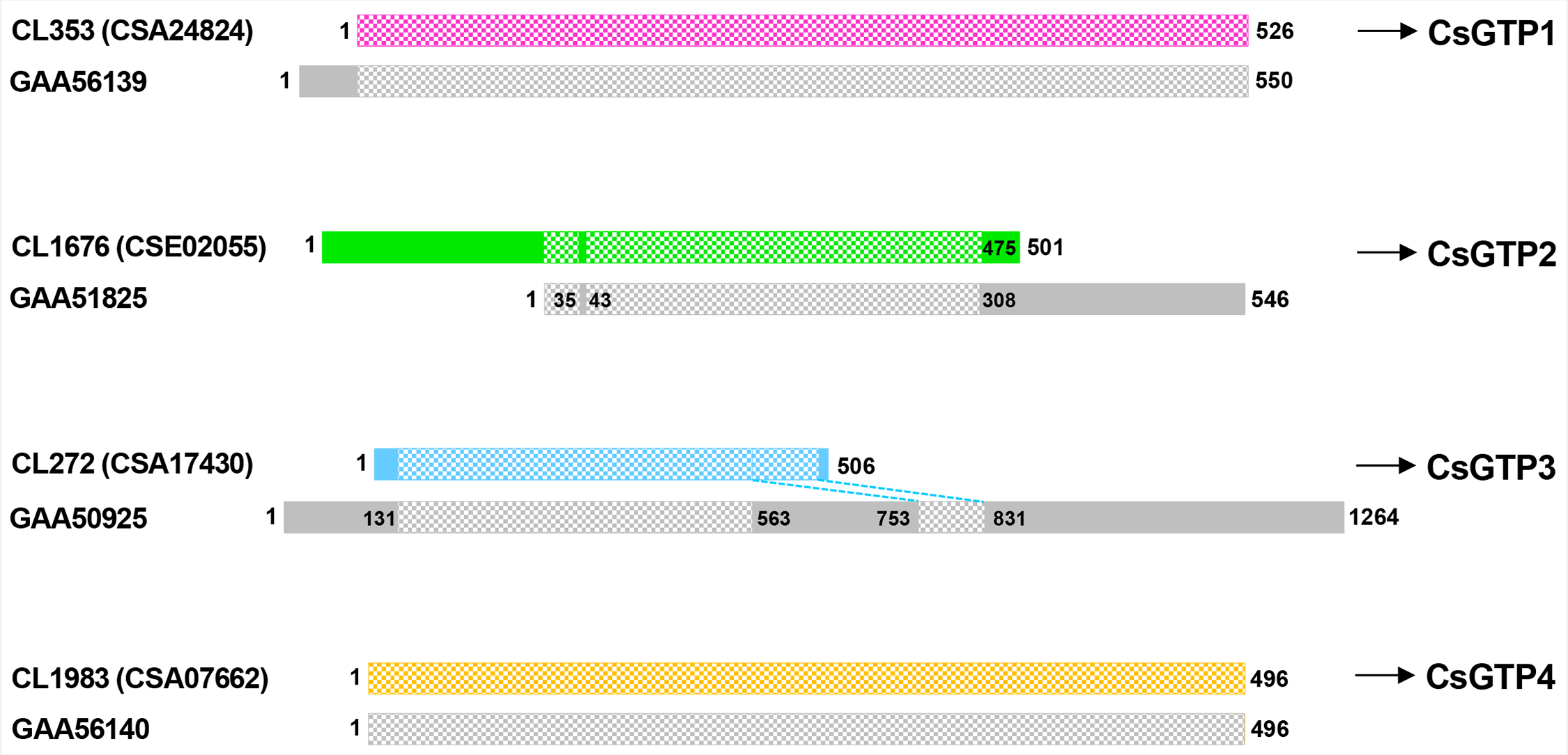

Supplement: S3 Fig — The hatched regions represent identical peptide sequences of the experimental and putative cDNAs, whereas the solid regions are different. GAAnnnnn is a putative CsGTP sequence obtained from GenBank (NCBI, NIH, USA). (TIF) [file pntd.0012315.s003.tif]

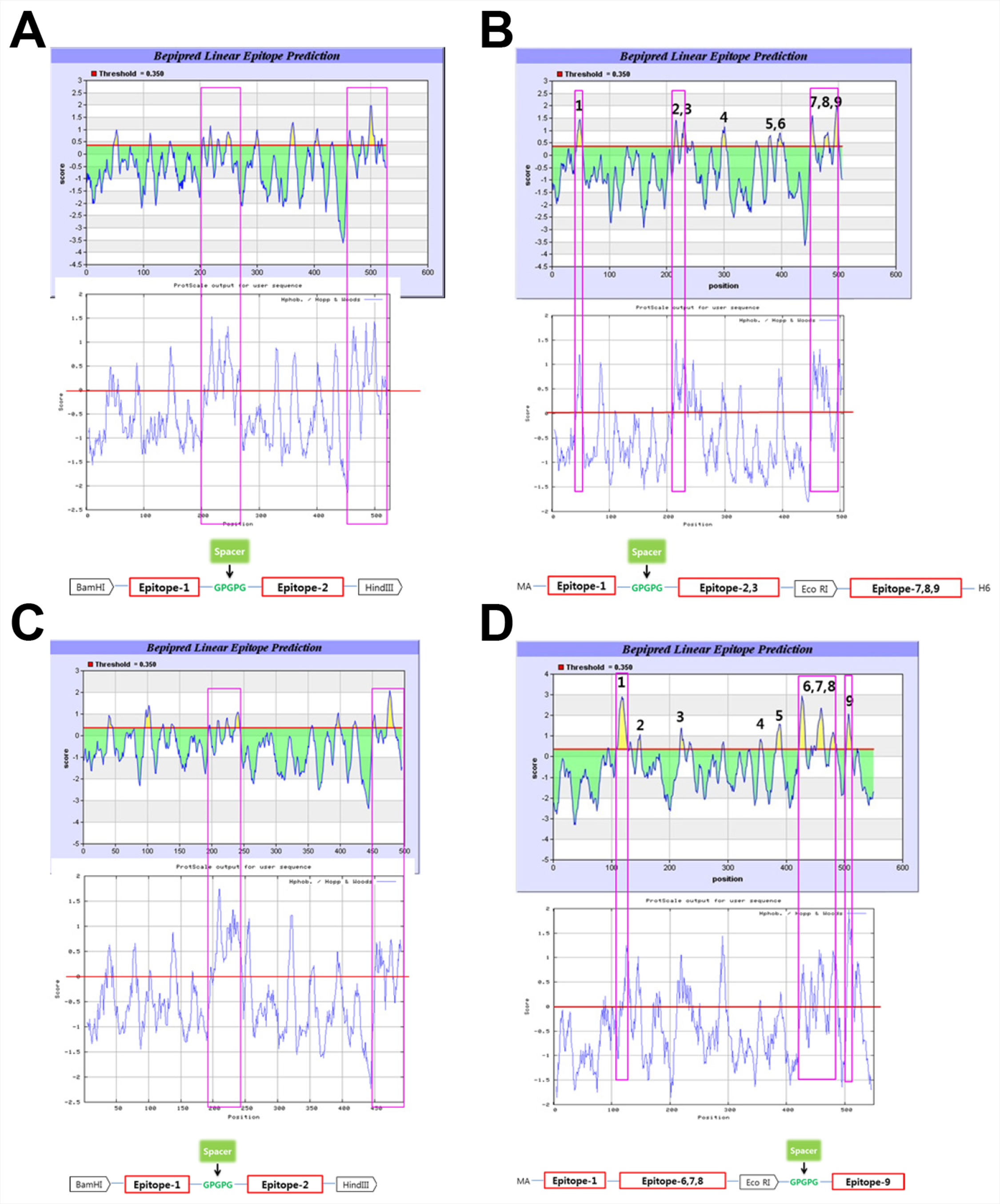

Supplement: S4 Fig — (A) CsGTP1; (B) CsGTP3; (C) CsGTP4; (D) CsSGLT. Pink boxes indicate B-cell epitopes matching hydrophilic regions. The two segments were bridged using the spacer peptide GPGPG, allowing the freedom of the segments. (TIF) [file pntd.0012315.s004.tif]

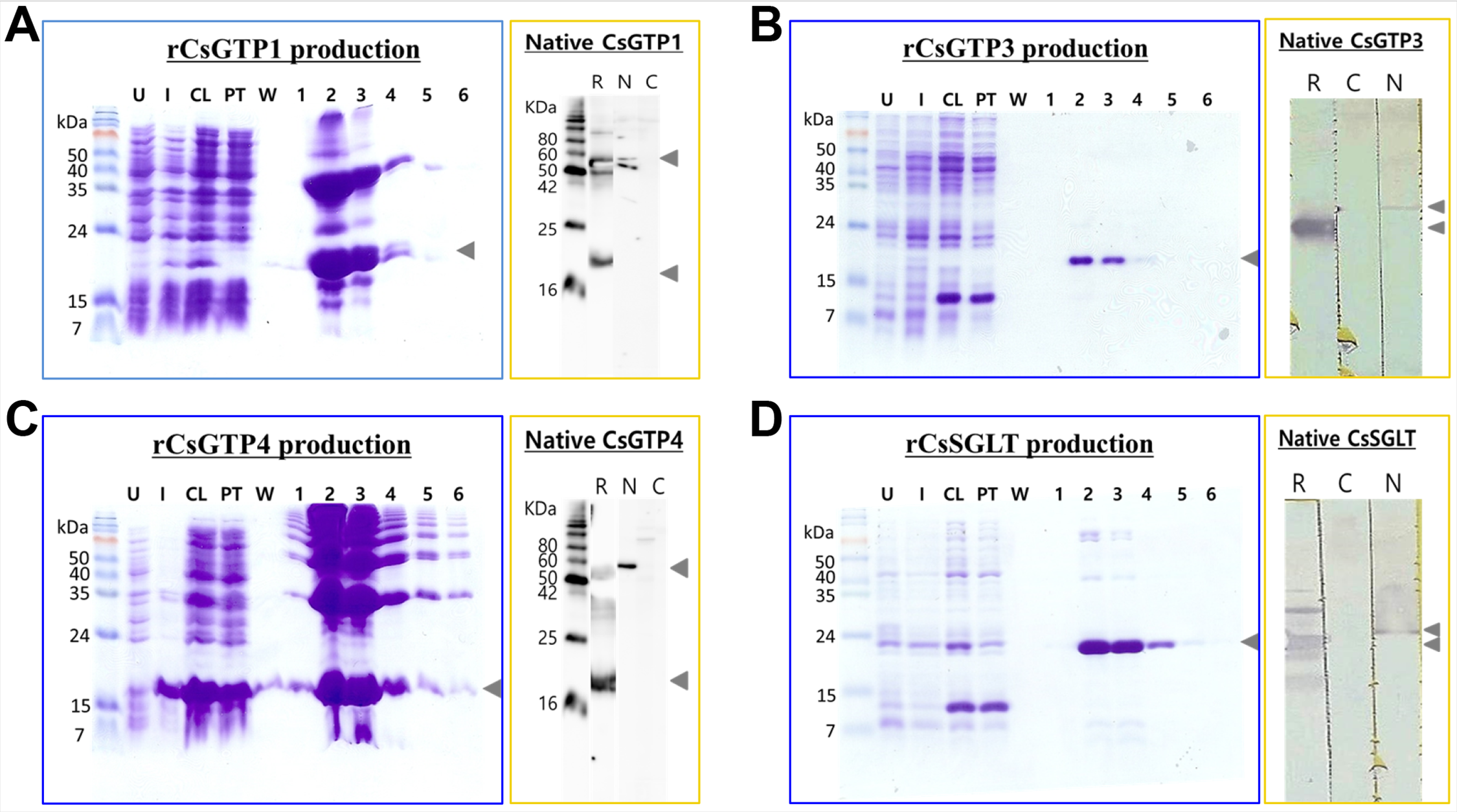

Supplement: S5 Fig — All recombinant proteins were purified by Ni-NTA column chromatography. Left panel: fractions were electrophoresed on SDS-PAGE gels and stained with Coomassie blue. Right panel: western blotting of the respective chimeric proteins using mouse immune sera. (A) Chimeric CsGTP1. (B) Chimeric CsGTP3. (C) Chimeric CsGTP4. (D) Chimeric CsSGLT. U: uninduced total fraction; I: induced total fraction; CL: clear lysate; PT: pass-through fraction; W: wash; lane 1–6, eluates from the Ni-NTA column; R: recombinant chimeric protein; N: native crude extract of C. sinensis; C: control. (TIF) [file pntd.0012315.s005.tif]

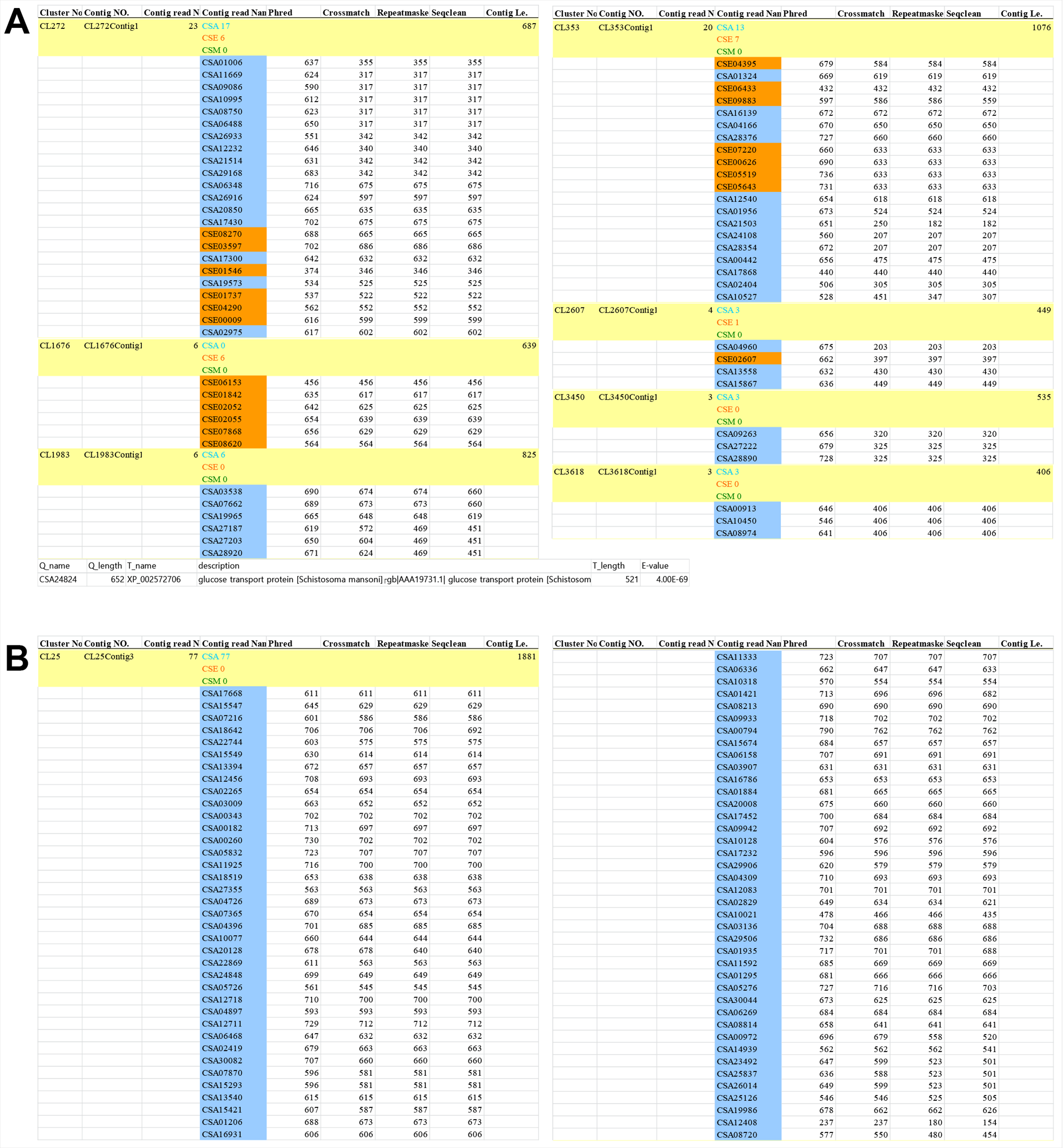

Supplement: S6 Fig — Contigs and expressed sequence tags (ESTs) encoding glucose transporters (A) and sodium glucose co-transporter (B) in the C. sinensis transcriptome database. The adult ESTs have a blue background, whereas the egg ESTs have an orange background. (TIF) [file pntd.0012315.s006.tif]

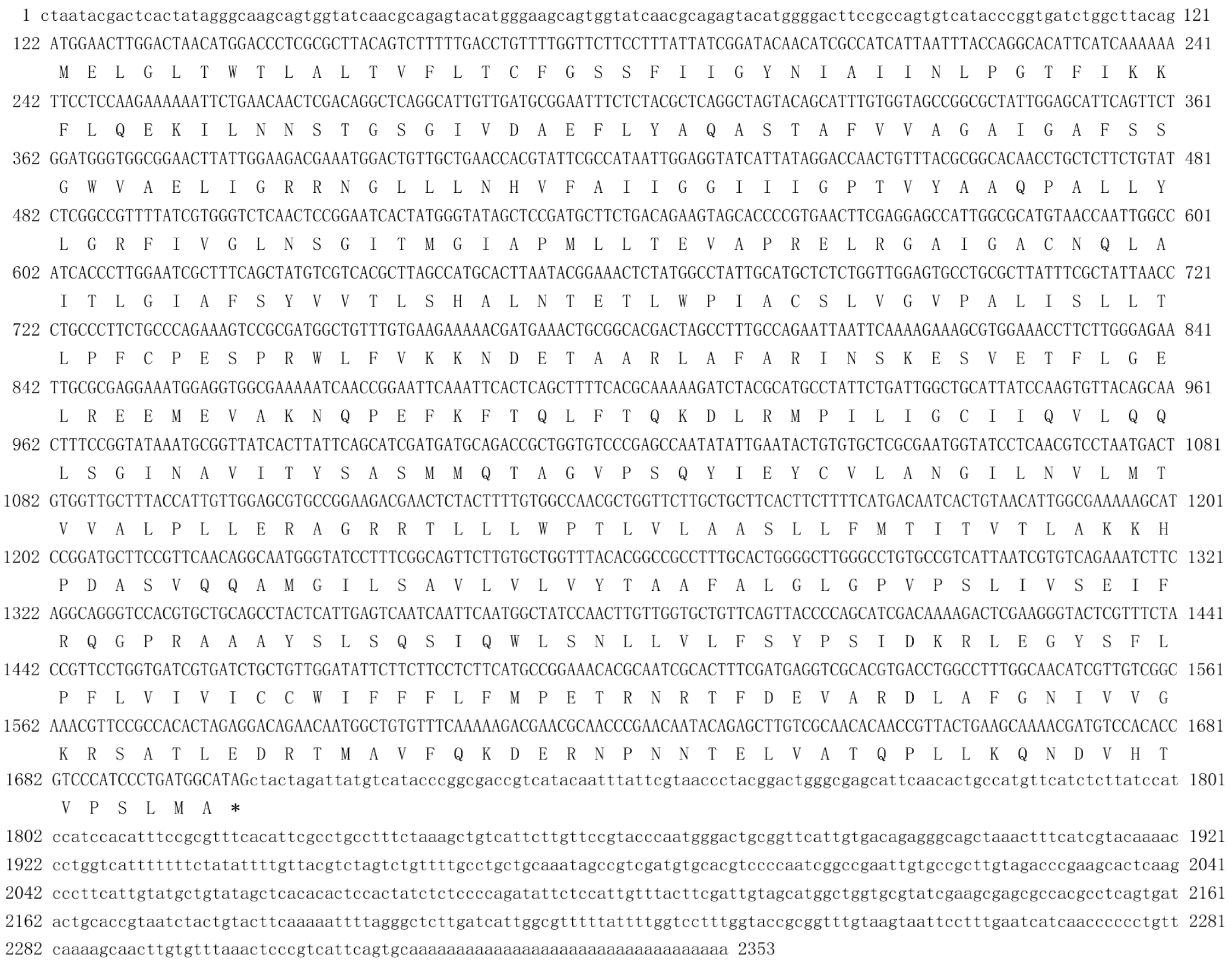

Supplement: S7 Fig — (TIF) [file pntd.0012315.s007.tif]

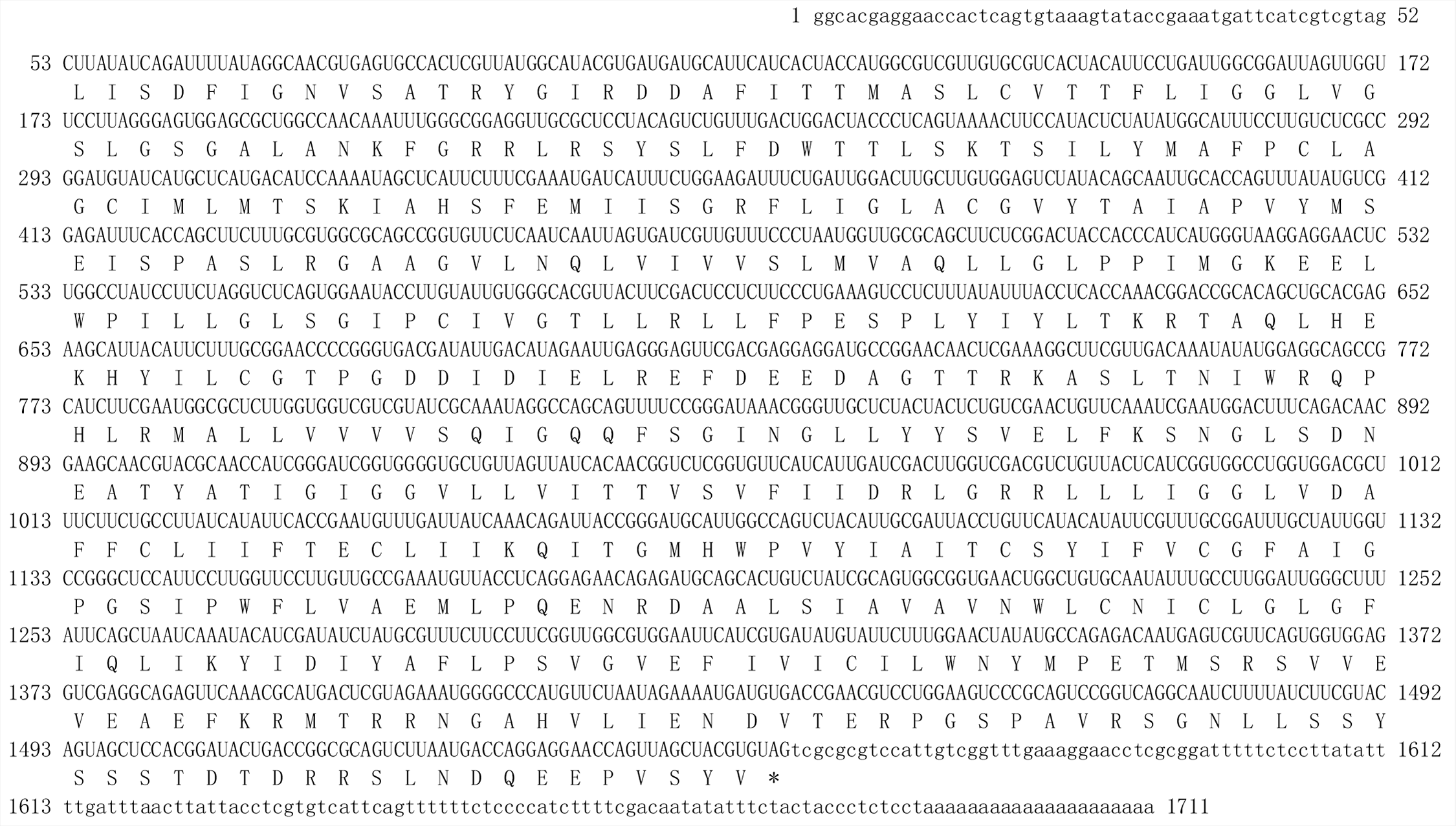

Supplement: S8 Fig — (TIF) [file pntd.0012315.s008.tif]

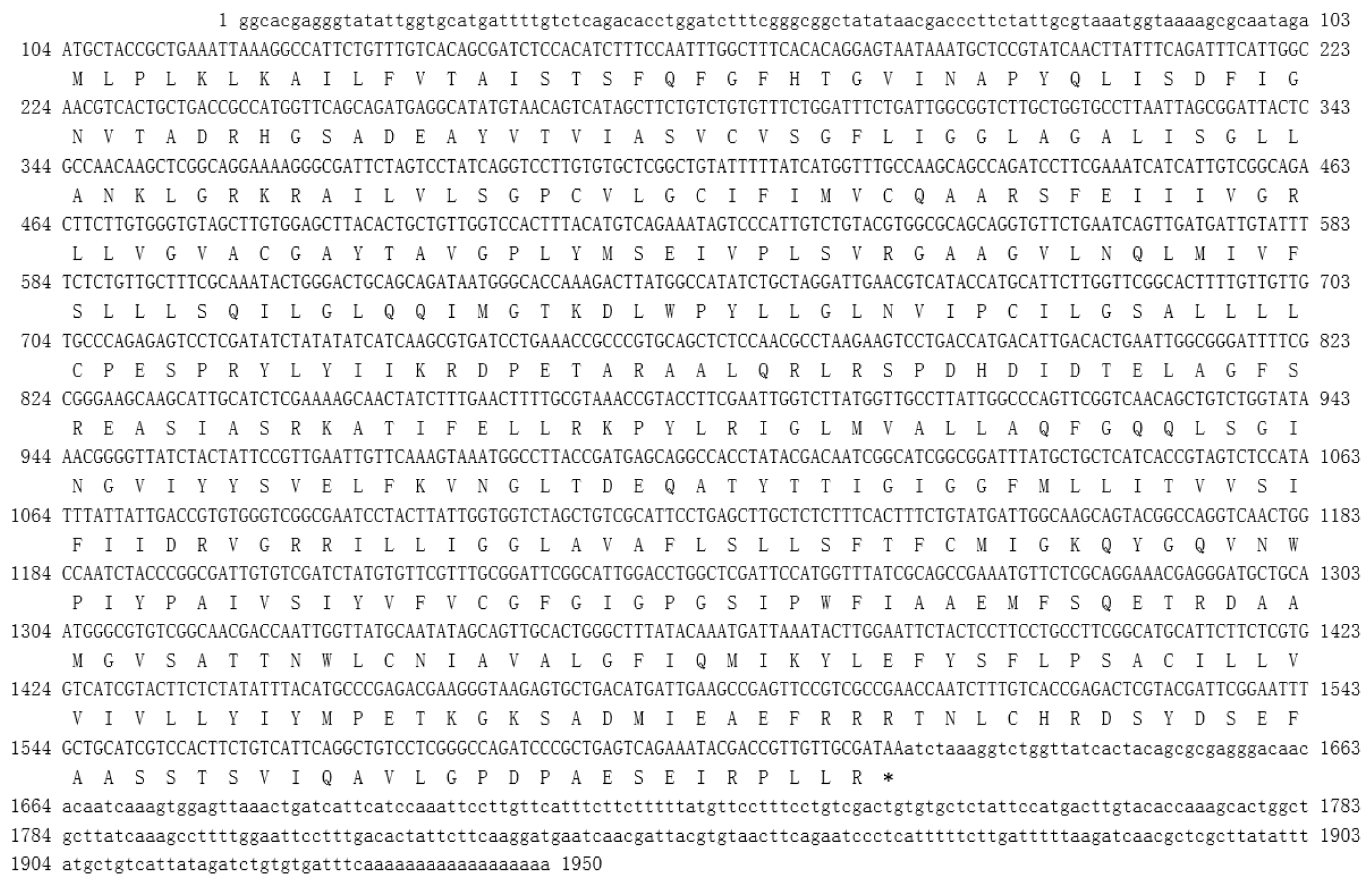

Supplement: S9 Fig — (TIF) [file pntd.0012315.s009.tif]

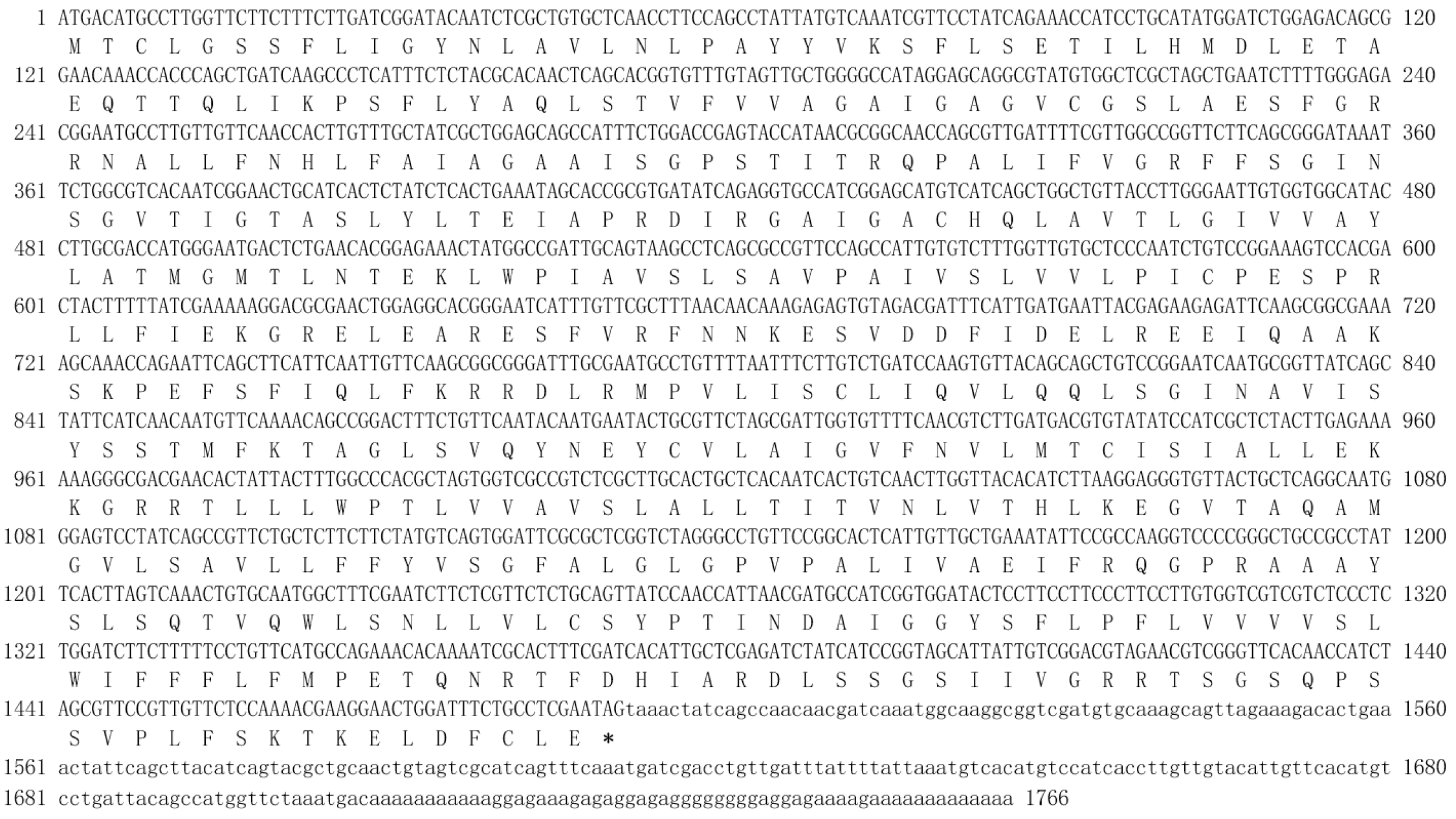

Supplement: S10 Fig — (TIF) [file pntd.0012315.s010.tif]

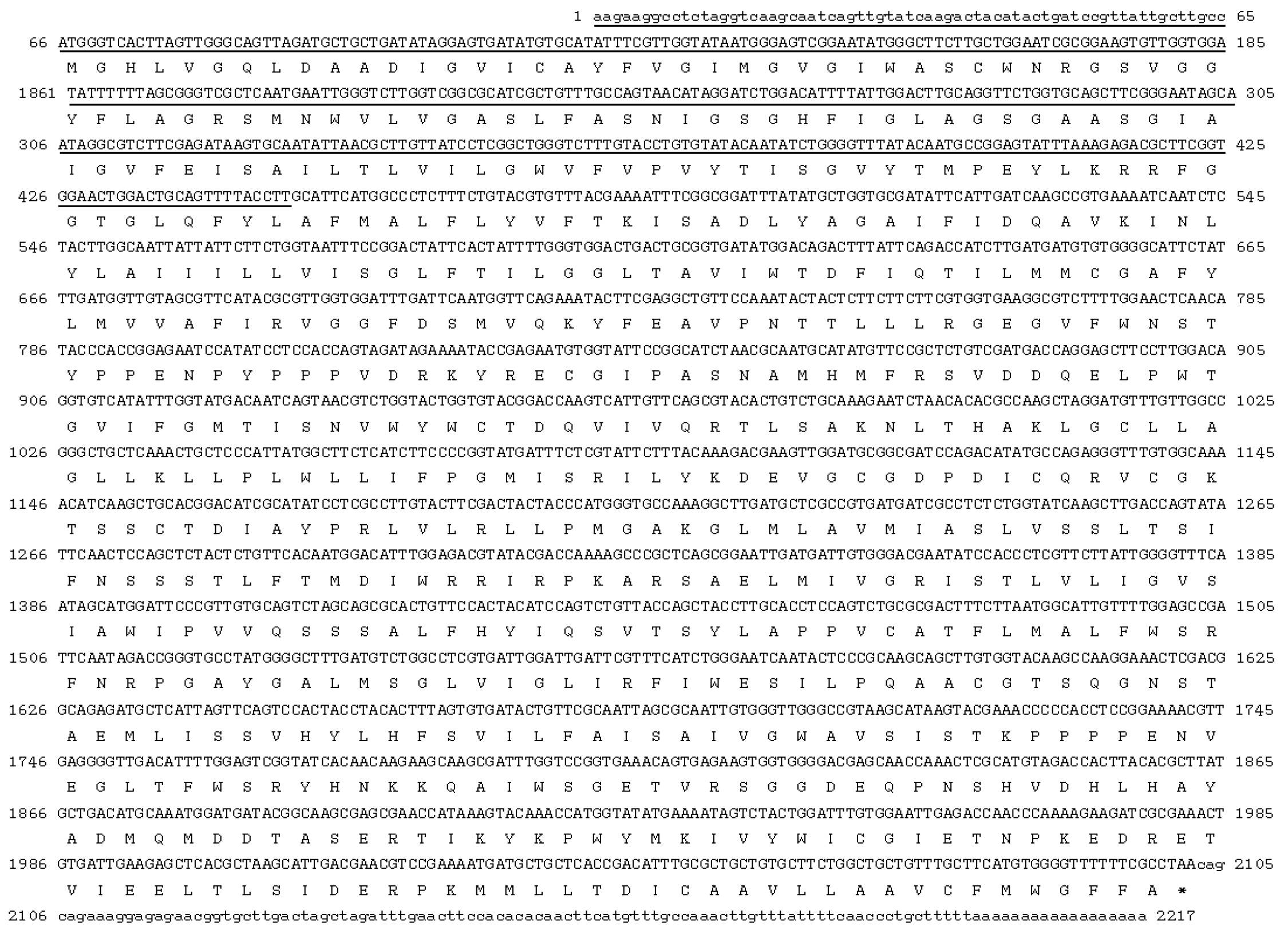

Supplement: S11 Fig — Nucleotide sequence obtained by 5′-RACE is underlined. (TIF) [file pntd.0012315.s011.tif]
