# Supplementary material for: Glucose transporters and sodium glucose co-transporters cooperatively import glucose into energy-demanding organs in carcinogenic liver fluke Clonorchis sinensis
Source: PLoS Negl Trop Dis. 2024 Jul 5;18(7):e0012315. doi: 10.1371/journal.pntd.0012315 (PMC11253919; doi:10.1371/journal.pntd.0012315)
Supplement: S1 Table — (DOCX) [file pntd.0012315.s012.docx]

**S1 Table. Expressed sequence tags (ESTs) encoding *C. sinensis* glucose transporter (CsGTP) and *C. sinensis* sodium glucose co-transporter (CsSGLT).**

| **EST** | **Description** | **e-value** | **Number of EST read** | | |
| --- | --- | --- | --- | --- | --- |
|  |  |  | **Adult** | **Metacercaria** | **Egg** |
| CL272Contig1 | Glucose transport protein | 7.10E-50 | 17 | 0 | 6 |
| CL1676Contig1 | Glucose transport protein | 4.80E-31 | 0 | 0 | 6 |
| CL353Contig1 | Glucose transport protein | 2.80E-51 | 13 | 0 | 7 |
| CL1983Contig1 | Glucose transport protein | 3.10E-67 | 6 | 0 | 0 |
| CL2607Contig1 | Glucose transport protein | 1.10E-52 | 3 | 0 | 1 |
| CL3450Contig1 | Glucose transport protein | 3.20E-33 | 3 | 0 | 0 |
| CL3618Contig1 | Glucose transport protein | 1.10E-29 | 3 | 0 | 0 |
| CSA24824 | Glucose transport protein | 2.10E-69 | 1 | - | - |
| CL25Contig3 | Sodium glucose co-transporter | 1.20E-107 | 77 | 0 | 0 |
