# Supplementary material for: Glucose transporters and sodium glucose co-transporters cooperatively import glucose into energy-demanding organs in carcinogenic liver fluke Clonorchis sinensis
Source: PLoS Negl Trop Dis. 2024 Jul 5;18(7):e0012315. doi: 10.1371/journal.pntd.0012315 (PMC11253919; doi:10.1371/journal.pntd.0012315)
Supplement: S2 Table — (DOCX) [file pntd.0012315.s013.docx]

**S2 Table. Secondary structural features of *C. sinensis* glucose transporter (CsGTP) subtypes: conserved residues and functional motifs.**

| No. | Motifs or residues | Conserved / functional | Location | Platyhelminth-specific | Class I GLUT-like |
| --- | --- | --- | --- | --- | --- |
|  |  |  |  | CsGTP1 and 4 | CsGTP2 and 3 |
| 1 | N-glycosylation site  (**N**xT/S) | Intracellular targeting, protein stability with high affinity to glucose [[1](#_ENREF_1),[2](#_ENREF_2)] | Loop1 | CsGTP1: **N**NS  CsGTP4: no | CsGTP2: **N**VS  CsGTP3: **N**VT |
| 2 | **N/E**xx**GRR/K** | Conserved | Loop2 | CsGTP1: **E**LI**GRR**  CsGTP4: **E**SF**GRR** | CsGTP2: **N**KF**GRR**  CsGTP3: **N**KL**GRK** |
| 3 | S/T**E**x_6_**RG** | **E** and/or **R** substitution greatly affect glucose transport activity; required for conformational change [[3](#_ENREF_3)] | Loop4 | CsGTP1: T**E**VAPREL**RG**  CsGTP4: T**E**IAPRDI**RG** | CsGTP2: S**E**ISPASL**RG**  CsGTP3: S**E**IVPLSV**RG** |
|  |  |  | Loop10 | **E**IFRQGP**R** | CsGTP2: **E**MLPQEN**R**  CsGTP3: **E**MFSQET**R** |
| 4 | **HQ**Lx_4_G  (Counterparts in GLUT1: H160, Q161) | **H**: binds in turn with O1 and O3 of glucose, work together with the latter Q to facilitate glucose entering inside [[4](#_ENREF_4)]  **Q**: facilitates glucose moving down to the inward gate without a change of orientation [[4](#_ENREF_4)]; critical for glucose transport [[5](#_ENREF_5),[6](#_ENREF_6)] | TM5 | CsGTP1: **HQ**LALTLG  CsGTP4: **HQ**LAVTLG | CsGTP2: N**Q**LVIVVS  CsGTP3: N**Q**LMIVFS |
| 5 | **W**P | Conserved | TM6 | **W**P | **W**P |
| 6 | **PESPR** | Conserved | Beginning of loop6 | **PESPR** | CsGTP2: **PESP**L  CsGTP3: **PESPR** |
| 7 | Q**QLS** | Interacting with the C1 of glucose and substrate selection at the exofacial binding site [[7](#_ENREF_7)]; the first Q contributes to sugar translocation and releasing inward [[4](#_ENREF_4)] | TM7 | Q**QLS** | CsGTP2: Q**QFS**  CsGTP3: Q**QLS** |
| 8 | **IN**A | After glucose enters the channel, the residues translocate glucose directly over the molecule and close the extracellular vestibule [[4](#_ENREF_4)] | TM7 | **IN**A | **IN**G |
| 9 | **Y**  (Counterparts in GLUT1: Y293) | Closing the exofacial site around C4 and C6 of glucose in the transport process [[8](#_ENREF_8)] | TM7 | CsGTP1: T**Y**  CsGTP4: S**Y** | **YY** |
| 10 | D**/E**xx**GRR/K** | **E**: mutagenesis fully abolishes glucose transport activity [[3](#_ENREF_3)]  **R**: conserved but not functional [[3](#_ENREF_3)] | Loop8 | CsGTP1: **E**RA**GRR**  CsGTP4: **E**KK**GRR** | CsGTP2: **D**RL**GRR**  CsGTP3: **D**RV**GRR** |
| 11 | **W**  (Counterparts in GLUT1: W388, W412) | **W**-TM10: forms hydrogen bond with glucose to help it enter inside [[4](#_ENREF_4)]; binding site of forskolin [[9](#_ENREF_9)] and cytochalasin B [[10](#_ENREF_10)]  **W**-TM11: cytochalasin B binding site [[7](#_ENREF_7)]; part of hexose binding site and critical for transport function [[11](#_ENREF_11),[12](#_ENREF_12)]; hydrophobic interaction with the C6 of glucose [[12](#_ENREF_12),[13](#_ENREF_13)] | TM10, TM11 | No W-TM10, W-TM11 | W-TM10, W-TM11 |
| 12 | **N** | Contributes to transport activity [[14](#_ENREF_14)]; binds with O4-glucose to help it move down to inner pocket [[4](#_ENREF_4)] | TM11 | **Q** | **N** |
| 13 | **PET**KG | Conserved | C-terminal tail | CsGTP1: **PET**RN  CsGTP4: **PET**QN | CsGTP2: **PET**MS  CsGTP3: **PETKG** |

**References**

1. Asano T, Katagiri H, Takata K, Lin JL, Ishihara H, Inukai K, et al. The role of *N*-glycosylation of GLUT1 for glucose transport activity. J Biol Chem. 1991; 266(36):24632-6.

2. Asano T, Takata K, Katagiri H, Ishihara H, Inukai K, Anai M, et al. The role of *N*-glycosylation in the targeting and stability of GLUT1 glucose transporter. FEBS Lett. 1993; 324(3):258-61.

3. Schurmann A, Doege H, Ohnimus H, Monser V, Buchs A, Joost HG. Role of conserved arginine and glutamate residues on the cytosolic surface of glucose transporters for transporter function. Biochemistry. 1997; 36(42):12897-902.

4. Park MS. Molecular dynamics simulations of the human glucose transporter GLUT1. PloS one. 2015; 10(4):e0125361.

5. Olsowski A, Monden I, Krause G, Keller K. Cysteine scanning mutagenesis of helices 2 and 7 in GLUT1 identifies an exofacial cleft in both transmembrane segments. Biochemistry. 2000; 39(10):2469-74.

6. Mueckler M, Weng W, Kruse M. Glutamine 161 of Glut1 glucose transporter is critical for transport activity and exofacial ligand binding. J Biol Chem. 1994; 269(32):20533-8.

7. Inukai K, Asano T, Katagiri H, Anai M, Funaki M, Ishihara H, et al. Replacement of both tryptophan residues at 388 and 412 completely abolished cytochalasin B photolabelling of the GLUT1 glucose transporter. Biochem J. 1994; 302:355-61.

8. Mori H, Hashiramoto M, Clark AE, Yang J, Muraoka A, Tamori Y, et al. Substitution of tyrosine 293 of GLUT1 locks the transporter into an outward facing conformation. J Biol Chem. 1994; 269(15):11578-83.

9. Schurmann A, Keller K, Monden I, Brown FM, Wandel S, Shanahan MF, et al. Glucose transport activity and photolabelling with 3-[^125^I]iodo-4-azidophenethylamido-7-*O*-succinyldeacetyl (IAPS)-forskolin of two mutants at tryptophan-388 and -412 of the glucose transporter GLUT1: dissociation of the binding domains of forskolin and glucose. Biochem J. 1993; 290:497-501.

10. Zhao FQ, Keating AF. Functional properties and genomics of glucose transporters. Curr Genomics. 2007; 8(2):113-28.

11. Katagiri H, Asano T, Shibasaki Y, Lin JL, Tsukuda K, Ishihara H, et al. Substitution of leucine for tryptophan 412 does not abolish cytochalasin B labeling but markedly decreases the intrinsic activity of GLUT1 glucose transporter. J Biol Chem. 1991; 266(12):7769-73.

12. Garcia JC, Strube M, Leingang K, Keller K, Mueckler MM. Amino acid substitutions at tryptophan 388 and tryptophan 412 of the HepG2 (Glut1) glucose transporter inhibit transport activity and targeting to the plasma membrane in *Xenopus* oocytes. J Biol Chem. 1992; 267(11):7770-6.

13. Mueckler M, Makepeace C. Analysis of transmembrane segment 10 of the Glut1 glucose transporter by cysteine-scanning mutagenesis and substituted cysteine accessibility. J Biol Chem. 2002; 277(5):3498-503.

14. Hruz PW, Mueckler MM. Cysteine-scanning mutagenesis of transmembrane segment 11 of the GLUT1 facilitative glucose transporter. Biochemistry. 2000; 39(31):9367-72.
