# Supplementary material for: Glucose transporters and sodium glucose co-transporters cooperatively import glucose into energy-demanding organs in carcinogenic liver fluke Clonorchis sinensis
Source: PLoS Negl Trop Dis. 2024 Jul 5;18(7):e0012315. doi: 10.1371/journal.pntd.0012315 (PMC11253919; doi:10.1371/journal.pntd.0012315)
Supplement: S3 Table — (DOCX) [file pntd.0012315.s014.docx]

**S3 Table. Conserved residues and characteristic functional motifs in *C. sinensis* sodium glucose co-transporter (CsSGLT).**

| No. | Motifs or residues | Conserved or functional | Location | CsSGLT | hSGLT1 | hSGLT2 |
| --- | --- | --- | --- | --- | --- | --- |
| 1 | **R**G**T**VGGx**FLAG**RSMx**W**x_3_**GAS** | Conserved [[1](#_ENREF_1)] | Loop1, TM2 | **R**G**S**VGGY**FLAG**RSMN**W**VLV**GAS** | **R**G**T**VGGF**FLAG**RSMV**W**WPI**GAS** | **R**G**T**VGGY**FLAG**RSMV**W**WPV**GAS** |
| 2 | N-glycosylation site  (**N**XT/S) | Conserved [[2](#_ENREF_2)] | Loop6 | **N**ST | **N**TT | **NIS** |
| 3 | **A, I, D**  (Counterparts in hSGLT1: A76, I79, D204) | Na2 binding site [[3](#_ENREF_3)] | TM2, loop5 | **A, I, D** | **A, I, D** | **A, I, D** |
| 4 | **N, H, E**  (Counterparts in hSGLT1: N79, H83, E102) | Na1 and glucose binding site [[3](#_ENREF_3)] | TM2, beginning of TM3 | **N, H, E** | **N, H, E** | **N, H, E** |
| 5 | **YW**  (Counterparts in hSGLT1: Y290, W291) | Na1 and glucose binding site [[3](#_ENREF_3)] | TM7 | **YW** | **YW** | **YW** |
| 6 | **K**  (Counterpart in hSGLT1: K321) | Glucose binding site [[3](#_ENREF_3)] | TM8 | **K** | **K** | **K** |
| 7 | S…**SS**  (Counterparts in hSGLT1: S389, **S**392, **S**393) | Na2 binding site [[3](#_ENREF_3)] | TM9 | S…**SS** | S…**SS** | A…**SS** |
| 8 | **Q**x_2_T  (Counterparts in hSGLT1: **Q**457x_2_T460) | **Q**: sugar binding residue in the hydrophilic channel; sugar moiety of phlorizin also interacts with this [[4](#_ENREF_4)] | Beginning of TM11 | **Q**x_2_T | **Q**x_2_T | **Q**x_2_S |

**References**

1. Wright EM, Loo DD, Hirayama BA, Turk E. Surprising versatility of Na^+^-glucose cotransporters: SLC5. Physiology (Bethesda). 2004; 19:370-6.

2. Wright EM, Turk E. The sodium/glucose cotransport family SLC5. Pflugers Arch. 2004; 447(5):510-8.

3. Loo DD, Jiang X, Gorraitz E, Hirayama BA, Wright EM. Functional identification and characterization of sodium binding sites in Na symporters. Proc Natl Acad Sci USA. 2013; 110(47):E4557-66.

4. Tyagi NK, Kumar A, Goyal P, Pandey D, Siess W, Kinne RK. D-Glucose-recognition and phlorizin-binding sites in human sodium/D-glucose cotransporter 1 (hSGLT1): a tryptophan scanning study. Biochemistry. 2007; 46(47):13616-28.
